# Brighter nights and darker days predict higher mortality risk: A prospective analysis of personal light exposure in >88,000 individuals

**DOI:** 10.1073/pnas.2405924121

**Published:** 2024-10-15

**Authors:** Daniel P. Windred, Angus C. Burns, Jacqueline M. Lane, Patrick Olivier, Martin K. Rutter, Richa Saxena, Andrew J. K. Phillips, Sean W. Cain

**Affiliations:** ^a^Flinders Health and Medical Research Institute (Sleep Health), Flinders University, Bedford Park, SA 5042, Australia; ^b^School of Psychological Sciences, Faculty of Medicine, Nursing and Health Sciences, Monash University, Melbourne, VIC 3800, Australia; ^c^Division of Sleep and Circadian Disorders, Brigham and Women’s Hospital, Boston, MA 02115; ^d^Division of Sleep Medicine, Harvard Medical School, Boston, MA 02115; ^e^Program in Medical and Population Genetics, Broad Institute, Cambridge, MA 02142; ^f^Center for Genomic Medicine, Massachusetts General Hospital, Boston, MA 02114; ^g^Action Lab, Department of Human-Centred Computing, Faculty of Information Technology, Monash University, Melbourne, VIC 3800, Australia; ^h^Centre for Biological Timing, Division of Endocrinology, Diabetes & Gastroenterology, School of Medical Sciences, Faculty of Biology, Medicine and Health, Manchester Academic Health Science Centre, University of Manchester, Manchester M13 9PL, United Kingdom; ^i^Diabetes, Endocrinology and Metabolism Centre, National Institute for Health and Care Research Manchester Biomedical Research Centre, Manchester University National Health Service Foundation Trust, Manchester M13 9WU, United Kingdom; ^j^Department of Anesthesia, Critical Care and Pain Medicine, Massachusetts General Hospital and Harvard Medical School, Boston, MA 02114

**Keywords:** light sensor, light at night, circadian disruption, longevity, cardiometabolic

## Abstract

Light enhances or disrupts circadian rhythms, depending on the timing of exposure. Circadian disruption contributes to poor health outcomes that increase mortality risk. Whether personal light exposure predicts mortality risk has not been established. We therefore investigated whether personal day and night light, and light patterns that disrupt circadian rhythms, predicted mortality risk. UK Biobank participants (N = 88,905, 62.4 ± 7.8 y, 57% female) wore light sensors for 1 wk. Day and night light exposures were defined by factor analysis of 24-h light profiles. A computational model of the human circadian pacemaker was applied to model circadian amplitude and phase from light data. Cause-specific mortality was recorded in 3,750 participants across a mean (±SD) follow-up period of 8.0 ± 1.0 y. Individuals with brighter day light had incrementally lower all-cause mortality risk (adjusted-HR ranges: 0.84 to 0.90 [50 to 70th light exposure percentiles], 0.74 to 0.84 [70 to 90th], and 0.66 to 0.83 [90 to 100th]), and those with brighter night light had incrementally higher all-cause mortality risk (aHR ranges: 1.15 to 1.18 [70 to 90th], and 1.21 to 1.34 [90 to 100th]), compared to individuals in darker environments (0 to 50th percentiles). Individuals with lower circadian amplitude (aHR range: 0.90 to 0.96 per SD), earlier circadian phase (aHR range: 1.16 to 1.30), or later circadian phase (aHR range: 1.13 to 1.20) had higher all-cause mortality risks. Day light, night light, and circadian amplitude predicted cardiometabolic mortality, with larger hazard ratios than for mortality by other causes. Findings were robust to adjustment for age, sex, ethnicity, photoperiod, and sociodemographic and lifestyle factors. Minimizing night light, maximizing day light, and keeping regular light–dark patterns that enhance circadian rhythms may promote cardiometabolic health and longevity.

## Significance

Exposure to brighter nights and darker days causes circadian disruption, which accompanies poor health outcomes that increase mortality risk. Whether personal day and night light exposure predicts mortality risk is not known. This study captured ~13 million hours of data from light sensors worn by ~89,000 individuals who were over 40 y of age. Those with brighter nights and darker days had higher risk of premature mortality, after accounting for sociodemographic and lifestyle factors. Avoiding night light and seeking day light may promote optimal health and longevity, and this recommendation is both accessible and cost-effective.

Circadian rhythm disruption accompanies a wide range of adverse health outcomes (1–3) that contribute to premature mortality. Light exposure at night disrupts circadian rhythms by shifting the timing (phase-shift) and weakening the signal (amplitude suppression) of the central circadian pacemaker in the hypothalamus (4–6), which orchestrates circadian rhythms throughout the body (7, 8). Experimental exposure to light at night causes premature mortality in animal models (9, 10). Human populations who are more likely to be exposed to light at night, including rotating shift-workers (11), evening types (12), and those with fragmented activity patterns (13, 14) have higher risks of premature mortality. Furthermore, population-scale studies have linked outdoor light at night with higher risk of all-cause mortality (15) and coronary heart disease (16), using satellite data. However, the relationship between personal night light exposure and risk of premature mortality in humans has not been investigated.

While night light disrupts circadian rhythms, day light stabilizes circadian phase (17), enhances circadian amplitude (18–20), and can reduce sensitivity of the circadian system to night light (21–23). Preventing circadian disruption by exposure to regular, bright day light may therefore protect against adverse health outcomes linked to circadian disruption (1–3). Cohort studies demonstrate that day light exposure protects against all-cause mortality (24, 25), cardiovascular disease mortality (26), myocardial infarction (25), stroke (27), and high blood pressure (28). However, these studies approximated day light exposure using self-reports (24, 26), satellite and ground-level measurements (27, 28), or by the presence or absence of skin cancers (25). Whether objective, personal day light exposure predicts premature mortality risk in humans has not been investigated.

Circadian disruption leads to cardiometabolic dysfunction and morbidity, which increases mortality risk (29). Experimental disruption of circadian rhythms alters blood glucose, insulin, cortisol, leptin, arterial pressure, and energy expenditure (30), and exposure to light patterns that can disrupt circadian rhythms predicts higher risk of type 2 diabetes (31). Myocardial infarction, stroke, hypertension, diabetes, and metabolic syndrome have higher incidence in rotating shift-workers (32–35). Cardiovascular risk factors, metabolic syndrome, and high BMI are also more often observed in evening types (30). In animal models, disruption of circadian rhythms with light produces profound cardiovascular disease, causing premature death due to cardiomyopathy, extensive fibrosis, and severely impaired contractility (36). However, no large-scale study has examined associations of individual-level light exposure with risk for premature mortality by cardiometabolic causes.

We characterized the relationship of personal light exposure with all-cause and cardiometabolic mortality risk in ~89,000 UK Biobank participants. Day light, night light, and modeled circadian phase and amplitude were derived from ~13 million hours of data recorded by wrist-worn light sensors, and participant mortality was captured by the National Health Service across a follow-up period of approximately 8 y. After accounting for age, sex, ethnicity, photoperiod, sociodemographic, and lifestyle factors, we found that higher risk of premature mortality was predicted by brighter nights, darker days, suppressed circadian amplitude, and early or late circadian phase.

## Results

We analyzed data from a subset of 88,905 UK Biobank participants who wore light sensors (Axivity AX3; Newcastle upon Tyne, UK) on their dominant wrist for 7 d under free-living conditions (2013 to 2016). Average daily light exposure profiles were extracted for each participant from their 7-d recording (see *SI Appendix*, Fig. S1 for average 24-h light exposure across the cohort). Factor analysis of average daily light exposure profiles revealed two distinct temporal clusters of light exposure, which were labeled as “day light” (07:30-20:30) and “night light” (00:30-06:00; see *SI Appendix*, section S1). Light exposures within day and night clusters were each split into four percentile groups, described in *SI Appendix*, Table S1.

Mean follow-up period was 7.99 ± 1.02 y between light recording and study endpoint of 22nd December, 2022, and total follow-up period was 9.55 y. Mortality records during follow-up were received by the UK Biobank from NHS Digital (England) and NHS Central Register (Scotland). Records included date of death and primary cause of death, diagnosed according to the ICD-10. All-cause mortality rate was 5.28 deaths per 1,000 person-years, including 3,750 all-cause and 798 cardiometabolic deaths.

Participants were 62.4 ± 7.8 y of age, 56.9% female, 97.0% white ethnicity, 62.1% employed, 43.4% with university education, 48.3% with other education, 6.8% current and 36.1% previous smokers, 84.1% from an urban postcode, and had median income range of £31,000 to 51,999, Townsend deprivation score of −1.8 ± 2.8, ≥1 weekly social activities (experienced by 72.8%), social visits most commonly experienced weekly (36.3%), alcohol consumption on 3.0 ± 2.5 d per week, and average physical activity of 28.1 ± 8.09 milli-g across weekly recordings. Participant characteristics within day light and night light percentile groups are provided in *SI Appendix*, Table S1.

### Night Light and Day Light Exposure Predicted Risk of All-Cause Mortality.

Exposure to incrementally brighter night light predicted higher risk of all-cause mortality, and exposure to incrementally brighter day light predicted lower risk of all-cause mortality (Table 1). Risk of mortality was estimated using adjusted Cox proportional hazards models that included both day and night light exposures, each split into 0 to 50th, 50 to 70th, 70 to 90th, and 90 to 100th percentile groups. Higher risk of all-cause mortality was observed for individuals in the 70 to 90th (aHR range: 1.15 to 1.18) and 90 to 100th (aHR range: 1.21 to 1.34) night light percentiles, compared to those with dark nights (0 to 50th percentiles). Lower risk of all-cause mortality was observed for individuals in the 50 to 70th (aHR range: 0.84 to 0.90), 70 to 90th (aHR range: 0.74 to 0.84), and 90 to 100th (aHR range: 0.66 to 0.83) day light percentiles, compared to individuals in the 0 to 50th day light percentiles. Number of all-cause deaths within light exposure percentile groups is provided in Table 1.

**Table 1.** Hazard ratios of all-cause mortality for Models 1 to 3, alongside percentage and number of deaths, according to percentile groups for day light and night light

		Light exposure percentile	% (N)	HR [95%CI]	*P*-value
Model 1	Night	0 to 50% (ref.)	4.09 (1,812)	–	–
N = 88,600		50 to 70%	3.88 (688)	1.02 [0.93 to 1.11]	0.69
		70 to 90%	4.44 (787)	1.17 [1.08 to 1.28]^*^	0.00022
		90 to 100%	5.01 (444)	1.34 [1.20 to 1.49]^*^	<0.0001
	Day	0 to 50% (ref.)	4.35 (1,925)	–	–
		50 to 70%	4.10 (726)	0.84 [0.77 to 0.92]^*^	0.00018
		70 to 90%	3.96 (701)	0.74 [0.67 to 0.82]^*^	<0.0001
		90 to 100%	4.28 (379)	0.66 [0.58 to 0.75]^*^	<0.0001
Model 2	Night	0 to 50% (ref.)	4.06 (1769)	–	–
N = 87,052		50 to 70%	3.87 (674)	1.02 [0.93 to 1.11]	0.74
		70 to 90%	4.44 (773)	1.18 [1.09 to 1.29]^*^	0.00012
		90 to 100%	4.94 (430)	1.31 [1.18 to 1.46]^*^	<0.0001
	Day	0 to 50% (ref.)	4.31 (1,878)	–	–
		50 to 70%	4.10 (713)	0.86 [0.79 to 0.94]^*^	0.0011
		70 to 90%	3.94 (686)	0.76 [0.69 to 0.84]^*^	<0.0001
		90 to 100%	4.24 (369)	0.68 [0.60 to 0.78]^*^	<0.0001
Model 3	Night	0 to 50% (ref.)	4.06 (1,737)	–	–
N = 85,562		50 to 70%	3.88 (664)	1.01 [0.92 to 1.10]	0.85
		70 to 90%	4.46 (763)	1.15 [1.06 to 1.25]^*^	0.0015
		90 to 100%	4.94 (423)	1.21 [1.08 to 1.35]^*^	0.00064
	Day	0 to 50% (ref.)	4.31 (1,844)	–	–
		50 to 70%	4.10 (702)	0.90 [0.83 to 0.99]^*^	0.031
		70 to 90%	3.95 (676)	0.84 [0.76 to 0.93]^*^	0.00075
		90 to 100%	4.27 (365)	0.83 [0.72 to 0.94]^*^	0.0042

Hazard ratios represent risks of all-cause mortality across light exposure percentile groups, compared to a reference light exposure group, for day and night light. Model 1 was adjusted for age, sex, ethnicity, and photoperiod; Model 2 was additionally adjusted for employment status, education, income, and deprivation; Model 3 was further adjusted for physical activity, smoking status, alcohol consumption, urbanicity, and social activity.

^*^P < 0.05.

Relationships of day and night light with all-cause mortality were robust to adjustment for potentially confounding factors across three hierarchical model levels: Model 1 was adjusted for age, sex, ethnicity, and photoperiod; Model 2 was additionally adjusted for employment status, education, income, and deprivation; and Model 3 was further adjusted for physical activity, smoking status, alcohol consumption, urbanicity, and social activity (Table 1). Relationships of day and night light with all-cause mortality were more highly attenuated in models with more comprehensive adjustments. Brighter night light and day light were robust predictors of higher and lower mortality risks, respectively, after additional adjustment of Model 3 for baseline vascular diagnoses (heart attack, stroke, angina), diabetes diagnosis, hypertension, high BMI, and high cholesterol ratio, and after exclusion of shift workers (*SI Appendix*, Table S3).

### Night Light and Day Light Exposure Predicted Risk of Cardiometabolic and Other-Cause Mortality.

Exposure to brighter night light predicted higher risks of cardiometabolic and other-cause mortality, and exposure to brighter day light predicted lower risks of cardiometabolic and other-cause mortality (Table 2). Risks of cause-specific mortality were estimated using proportional subhazards models for competing-risks (37), adjusted across three hierarchical levels, and including both day and night light exposures, as described above. Individuals in the 70 to 90th and 90 to 100th night light percentiles had, respectively, aHR ranges of 1.22 to 1.26 (70 to 90%) and 1.33 to 1.46 (90 to 100%) for cardiometabolic mortality, and aHR ranges of 1.13 to 1.15 (70 to 90%) and 1.17 to 1.30 (90 to 100%) for other-cause mortality, compared to those with dark nights (0 to 50th percentiles). Individuals in the 50 to 70th, 70 to 90th, and 90 to 100th day light percentiles had, respectively, aHR ranges of 0.79 to 0.84 (50 to 70%), 0.77 to 0.91 (70 to 90%), and 0.61 to 0.76 (90 to 100%) for cardiometabolic mortality, and aHR ranges of 0.86-0.92 (50 to 70%), 0.74 to 0.84 (70 to 90%), and 0.68 to 0.86 (90 to 100%) for other-cause mortality, compared to those in the 0 to 50th day light percentiles. Relationships of cardiometabolic and other-cause mortality with day light exposure were attenuated in Model 3, such that only the 90 to 100th day light percentiles remained statistically significant predictors of both mortality causes. Day light and night light predicted cardiometabolic mortality with larger hazard ratios than for other-cause mortality. Number of deaths by cardiometabolic causes and other causes, split by light exposure percentile groups, is provided in Table 2.

**Table 2.** Hazard ratios of cause-specific mortality for Models 1 to 3, alongside percentage and number of deaths, according to percentile groups for day light and night light

			Cardiometabolic mortality			Other-cause mortality		
		Light exposure percentile	% (N)	HR [95%CI]	*P*-value	% (N)	HR [95%CI]	*P*-value
Model 1	Night	0 to 50% (ref.)	0.84 (373)	–	–	3.22 (1,426)	–	–
		50 to 70%	0.85 (150)	1.08 [0.89 to 1.31]	0.22	3.01 (534)	1.00 [0.91 to 1.11]	0.48
		70 to 90%	0.97 (172)	1.25 [1.04 to 1.49]^*^	0.0091	3.43 (608)	1.15 [1.04 to 1.26]^*^	0.0025
		90 to 100%	1.12 (99)	1.46 [1.16 to 1.82]^*^	0.00051	3.86 (342)	1.30 [1.15 to 1.47]^*^	<0.0001
	Day	0 to 50% (ref.)	0.93 (411)	–	–	3.38 (1,497)	–	–
		50 to 70%	0.82 (146)	0.79 [0.65 to 0.96]^*^	0.0099	3.23 (573)	0.86 [0.78 to 0.95]^*^	0.0015
		70 to 90%	0.90 (159)	0.77 [0.62 to 0.95]^*^	0.0084	3.05 (541)	0.74 [0.66 to 0.83]^*^	<0.0001
		90 to 100%	0.88 (78)	0.61 [0.46 to 0.80]^*^	0.00027	3.38 (299)	0.68 [0.59 to 0.79]^*^	<0.0001
Model 2	Night	0 to 50% (ref.)	0.84 (365)	–	–	3.20 (1,391)	–	–
		50 to 70%	0.86 (150)	1.10 [0.90 to 1.33]	0.17	2.99 (520)	0.99 [0.90 to 1.10]	0.45
		70 to 90%	0.97 (169)	1.26 [1.05 to 1.52]^*^	0.0068	3.43 (597)	1.15 [1.05 to 1.27]^*^	0.0019
		90 to 100%	1.11 (97)	1.45 [1.16 to 1.83]^*^	0.00064	3.79 (330)	1.27 [1.13 to 1.44]^*^	<0.0001
	Day	0 to 50% (ref.)	0.92 (402)	–	–	3.35 (1,459)	–	–
		50 to 70%	0.83 (145)	0.81 [0.66 to 0.99]^*^	0.018	3.22 (561)	0.88 [0.79 to 0.97]^*^	0.0058
		70 to 90%	0.91 (159)	0.80 [0.64 to 0.99]^*^	0.019	3.02 (526)	0.76 [0.68 to 0.85]^*^	<0.0001
		90 to 100%	0.86 (75)	0.61 [0.46 to 0.82]^*^	0.00044	3.35 (292)	0.72 [0.62 to 0.83]^*^	<0.0001
Model 3	Night	0 to 50% (ref.)	0.84 (360)	–	–	3.19 (1,364)	–	–
		50 to 70%	0.86 (148)	1.09 [0.90 to 1.32]	0.2	2.99 (512)	0.99 [0.89 to 1.09]	0.41
		70 to 90%	0.97 (166)	1.22 [1.01 to 1.47]^*^	0.019	3.45 (590)	1.13 [1.02 to 1.24]^*^	0.0091
		90 to 100%	1.12 (96)	1.33 [1.06 to 1.68]^*^	0.0069	3.79 (324)	1.17 [1.03 to 1.32]^*^	0.0064
	Day	0 to 50% (ref.)	0.93 (397)	–	–	3.34 (1,430)	–	–
		50 to 70%	0.82 (141)	0.84 [0.69 to 1.03]	0.051	3.24 (554)	0.92 [0.83 to 1.02]	0.064
		70 to 90%	0.92 (158)	0.91 [0.73 to 1.12]	0.19	3.02 (517)	0.84 [0.75 to 0.94]^*^	0.0011
		90 to 100%	0.86 (74)	0.76 [0.56 to 1.02]^*^	0.033	3.38 (289)	0.86 [0.74 to 0.99]^*^	0.02

Hazard ratios represent risks of cardiometabolic and other-cause mortality across light exposure percentile groups, compared to a reference light exposure group, for day and night light. Model 1 was adjusted for age, sex, ethnicity, and photoperiod; Model 2 was additionally adjusted for employment status, education, income, and deprivation; Model 3 was further adjusted for physical activity, smoking status, alcohol consumption, urbanicity, and social activity.

^*^P < 0.05.

### Time-of-Day Association of Light Exposure with Mortality Risk.

**Fig. 1.**
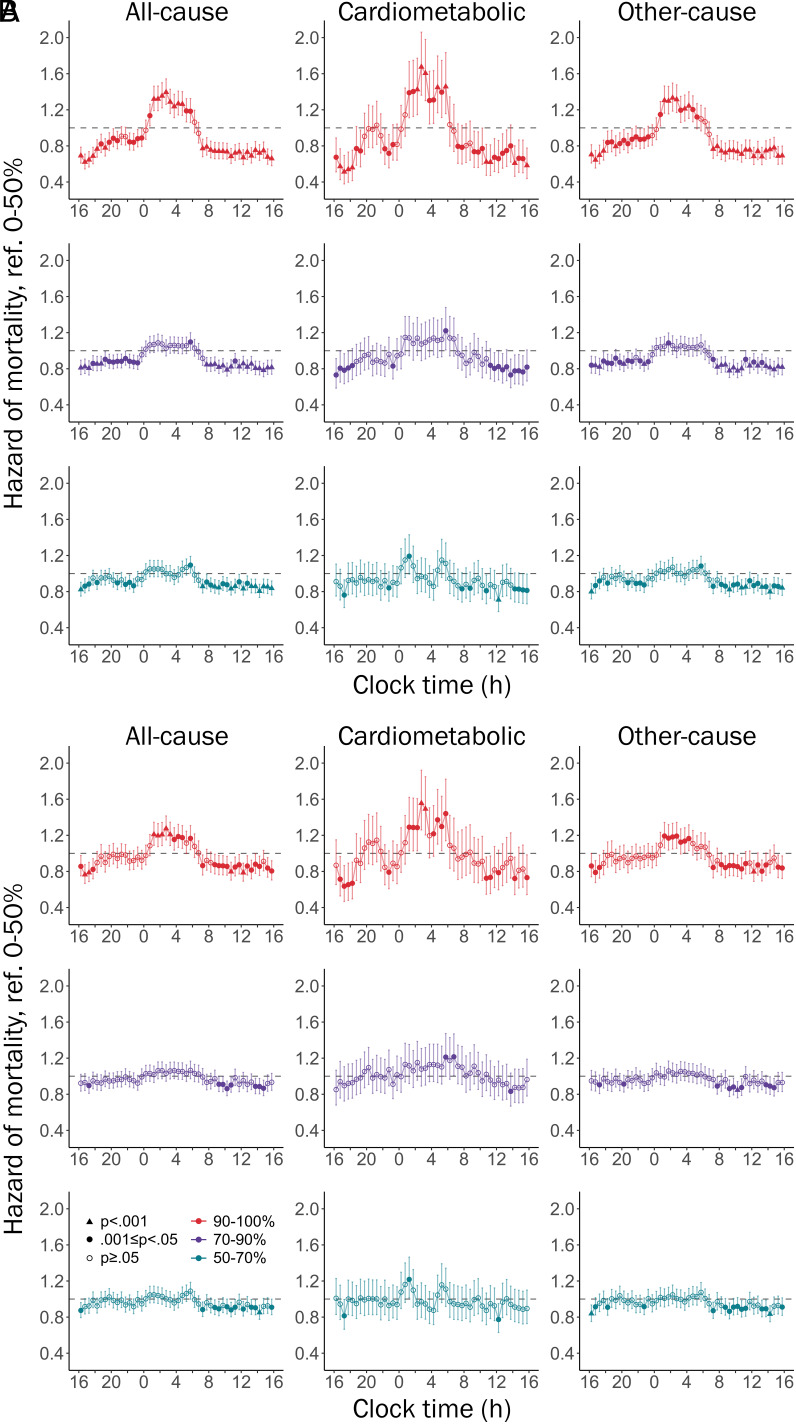
Hazard ratios [95%CI] of all-cause, cardiometabolic, and other-cause mortality for light exposures across 24 h, including Model 1 (*A*) and Model 3 (*B*). Separate models were implemented for each half-hour clock time interval, with each model including 50 to 70%, 70 to 90%, and 90 to 100% light percentile groups referenced against 0 to 50%. Bonferroni correction for multiple comparisons required *P* < 0.001 for statistical significance. For example, in column one of Panel *A*, individuals in the 90 to 100th percentiles of light exposure at 04:00 had a significantly higher risk of all-cause mortality than those in the 0 to 50th percentiles (HR = 1.27), whereas individuals in the 50 to 70th percentiles and 70 to 90th percentiles at 04:00 did not have a significantly higher mortality risk. See *SI Appendix*, Fig. S2 for mortality risk by 24-h light exposures adjusted for Model 2 covariates.

Risk of mortality was estimated for light exposures across 24 h, using 48 Cox models corresponding to half-hour clock time intervals, adjusted for multiple comparisons (Bonferroni, *P* < 0.001; see Fig. 1 and *SI Appendix*, Fig. S2). Light exposures within each half-hour interval were split into percentile groups, and models were adjusted across three hierarchical levels, as described above. Exposure to the brightest 10% of lighting environments in half-hour intervals between 01:00 and 06:00 predicted an 11 to 39% higher risk of all-cause mortality, and exposure between 08:30 and 18:00 predicted a 9 to 38% lower risk of all-cause mortality, compared to the darkest 0 to 50th percentiles. Exposure to the brightest 10% of environments between 01:00 and 06:00 predicted a 20 to 67% higher risk of mortality by cardiometabolic causes. The peak hazard ratio for cardiometabolic mortality (56 to 67% higher risk between 02:30 and 03:00) was greater than the peak hazard ratio for mortality by other causes (18 to 33% higher risk between 02:00 and 02:30).

### Lower Circadian Amplitude and Earlier or Later Circadian Phase Predicted Higher Risk of Mortality.

To model circadian amplitude and phase, the continuously recorded light data were input to a mathematical model (38) that approximates the response of human photoreceptors (represented by a dynamic stimulus processor) and the central circadian pacemaker (represented by a limit cycle oscillator) to light exposure. This model has been applied in various populations to predict the state of the human circadian clock from light data (39). Light modulates pacemaker phase and amplitude, with the response dependent on the state of the model at the time of light exposure. Light in the early and late biological night delays and advances circadian phase, respectively, and light near the middle of the biological night suppresses circadian amplitude. We used this modeling approach to extract average circadian phase, intraindividual variability in phase, and mean, minimum, and maximum circadian amplitudes across each participant’s 7-d light recording. These circadian variables were included as predictors of mortality in Cox models, adjusted across three hierarchical levels as described above.

Higher circadian amplitude predicted lower risks of all-cause and cardiometabolic mortality across Models 1 to 3 (Table 3). Lower mortality risk was observed with higher mean circadian amplitude (all-cause aHR range: 0.93 to 0.96 per SD; cardiometabolic aHR range: 0.88 to 0.92), higher minimum amplitude (all-cause aHR range: 0.94 to 0.96; cardiometabolic aHR range: 0.87 to 0.93), and higher maximum amplitude (all-cause aHR range: 0.90 to 0.94; cardiometabolic aHR range: 0.90 to 0.92). Higher minimum amplitude predicted lower other-cause mortality risk across Models 1 to 3 (aHR range: 0.91 to 0.95), and higher mean and maximum circadian amplitude predicted lower other-cause risks across Models 1 to 2 (aHR ranges: 0.95 to 0.95 and 0.96 to 0.96, respectively). Early and late circadian phase quintiles predicted higher risks of all-cause mortality (aHR ranges: 1.16 to 1.30 [0 to 20%] and 1.13 to 1.20 [80 to 100%]) and other-cause mortality (aHR ranges: 1.14 to 1.27 [0 to 20%] and 1.13 to 1.20 [80 to 100%]), compared to the quintile centered at the sample’s circular mean phase, across Models 1 to 3. Early circadian phase predicted cardiometabolic mortality across Models 1 to 3 (aHR range: 1.27 to 1.43 [0 to 20%]). Intraindividual variability in circadian phase was not related to mortality. Relationships of mortality risk with circadian phase and amplitude were consistent with the relationships of mortality risk with light exposure between 0:30 and 06:00, when light would be expected to significantly delay, advance, or suppress the circadian rhythms of most individuals. Relationships of mortality risk with modeled circadian rhythms were generally robust to adjustment for baseline cardiometabolic health and exclusion of shift workers (*SI Appendix*, Table S3). Numbers of all-cause, cardiometabolic, and noncardiometabolic deaths across the range of each circadian variable are reported *SI Appendix*, Table S4. Early and late modeled circadian phase were predictive of early and late midsleep timing, respectively, on weekends and weekdays (*SI Appendix*, Table S5).

**Table 3.** Hazard ratios of all-cause, cardiometabolic, and other-cause mortality for modeled circadian rhythm metrics

		All-cause mortality		Cardiometabolic mortality		Other-cause mortality	
		HR [95%CI]	*P*-value	HR [95%CI]	*P*-value	HR [95%CI]	*P*-value
Model 1	Mean Amplitude	0.93 [0.90 to 0.96]^*^	<0.0001	0.88 [0.82 to 0.95]^*^	0.00021	0.95 [0.92 to 0.99]^*^	0.006
	Min. Amplitude	0.95 [0.92 to 0.98]^*^	0.00035	0.87 [0.80 to 0.94]^*^	0.00031	0.91 [0.88 to 0.95]^*^	<0.0001
	Max. Amplitude	0.90 [0.87 to 0.93]^*^	<0.0001	0.91 [0.85 to 0.96]^*^	0.00078	0.96 [0.93 to 1.00]^*^	0.017
	Phase Variability	1.01 [0.98 to 1.04]	0.42	0.99 [0.94 to 1.05]	0.4	1.02 [0.99 to 1.05]	0.14
	Mean phase						
	0 to 20%	1.30 [1.17 to 1.44]^*^	<0.0001	1.43 [1.14 to 1.79]^*^	0.0011	1.27[ 1.12 to 1.42]^*^	<0.0001
	20 to 40%	1.13 [1.02 to 1.27]^*^	0.023	1.25 [0.98 to 1.59]^*^	0.035	1.10 [0.97 to 1.24]	0.071
	40 to 60% (ref.)	–	–	–	–	–	–
	60 to 80%	1.07 [0.96 to 1.20]	0.22	1.21 [0.95 to 1.54]	0.059	1.04 [0.92 to 1.18]	0.26
	80 to 100%	1.20 [1.07 to 1.34]^*^	0.0016	1.19 [0.92 to 1.52]	0.091	1.20 [1.05 to 1.36]^*^	0.003
Model 2	Mean Amplitude	0.93 [0.90 to 0.96]^*^	<0.0001	0.88 [0.82 to 0.95]^*^	0.00022	0.95 [0.91 to 0.99]^*^	0.0034
	Min. Amplitude	0.94 [0.91 to 0.97]^*^	<0.0001	0.88 [0.81 to 0.96]^*^	0.0013	0.92 [0.88 to 0.96]^*^	<0.0001
	Max. Amplitude	0.90 [0.87 to 0.94]^*^	<0.0001	0.90 [0.85 to 0.96]^*^	0.00045	0.96 [0.92 to 0.99]^*^	0.0077
	Phase variability	1.01 [0.98 to 1.04]	0.43	0.99 [0.94 to 1.05]	0.4	1.02 [0.99 to 1.04]	0.15
	Mean phase						
	0 to 20%	1.21 [1.09 to 1.35]^*^	0.00041	1.33 [1.06 to 1.68]^*^	0.0079	1.18 [1.05 to 1.33]^*^	0.0032
	20 to 40%	1.11 [1.00 to 1.24]	0.054	1.21 [0.95 to 1.54]	0.06	1.08 [0.95 to 1.22]	0.11
	40 to 60% (ref.)	–	–	–	–	–	–
	60 to 80%	1.08 [0.97 to 1.21]	0.17	1.24 [0.97 to 1.57]^*^	0.044	1.05 [0.92 to 1.19]	0.24
	80 to 100%	1.20 [1.07 to 1.35]^*^	0.0014	1.20 [0.93 to 1.54]	0.081	1.20 [1.05 to 1.37]^*^	0.0029
Model 3	Mean Amplitude	0.96 [0.92 to 0.99]^*^	0.0083	0.92 [0.86 to 0.98]^*^	0.0076	0.97 [0.94 to 1.01]	0.076
	Min. Amplitude	0.96 [0.93 to 0.99]^*^	0.0052	0.93 [0.86 to 1.01]^*^	0.04	0.95 [0.91 to 0.99]^*^	0.007
	Max. Amplitude	0.94 [0.91 to 0.98]^*^	0.0011	0.92 [0.87 to 0.98]^*^	0.0043	0.97 [0.94 to 1.01]	0.056
	Phase variability	1.01 [0.98 to 1.03]	0.56	0.99 [0.94 to 1.05]	0.37	1.01 [0.98 to 1.04]	0.19
	Mean phase						
	0 to 20%	1.16 [1.04 to 1.29]^*^	0.0066	1.27 [1.00 to 1.61]^*^	0.023	1.14 [1.00 to 1.28]^*^	0.021
	20 to 40%	1.11 [0.99 to 1.24]	0.065	1.20 [0.94 to 1.53]	0.073	1.08 [0.95 to 1.22]	0.12
	40 to 60% (ref.)	–	–	–	–	–	–
	60 to 80%	1.07 [0.96 to 1.20]	0.22	1.22 [0.96 to 1.56]	0.055	1.04 [0.92 to 1.18]	0.26
	80 to 100%	1.13 [1.00 to 1.26]^*^	0.042	1.10 [0.85 to 1.41]	0.24	1.13 [0.99 to 1.29]^*^	0.031

Hazard ratios represent difference in mortality hazard per SD increase in each circadian metric for mean, min., and max. amplitude, and phase variability. Hazard ratios for mean phase represent hazard of each percentile group relative to the 40 to 60% reference group, centered at the population mean phase (03:50). Phase ranges relative to sample mean for each percentile group were −12 to −1.16 h (0 to 20%), −1.16 to −0.40 h (20 to 40%), 0.40 to 1.16 h (60 to 80%), and 1.16 to 12 h (80 to 100%). Model 1 was adjusted for age, sex, ethnicity, and photoperiod; Model 2 was additionally adjusted for employment status, education, income, and deprivation; Model 3 was further adjusted for physical activity, smoking status, alcohol consumption, urbanicity, and social activity.

^*^P < 0.05.

### Sleep Duration and Sleep Efficiency in Light-Mortality Relationships.

Sleep disruption is linked to higher risk of premature mortality (40–43) and sleep shares a bidirectional relationship with light exposure. We therefore assessed whether sleep explained the observed relationships of light exposure and modeled circadian disruption with premature mortality risk. Sleep–wake state was estimated from participants’ 7-d accelerometer recordings (Axivity AX3 device), as per our previous work (44, 45). We extracted average sleep efficiency (percentage of sleep between onset and offset times; median [IQR] = 0.90 [0.87 to 0.93]) and identified short sleepers (<6 h; 19.6% of individuals) and long sleepers (>9 h; 1.3% of individuals). Models predicting mortality risk from day and night light, and from modeled circadian rhythms, were adjusted for each sleep variable.

The relationship of night light with all-cause mortality in Model 3 was attenuated with the inclusion of short sleep (aHR [95%CI] for 70 to 90%: 1.11 [1.02 to 1.22]; 90 to 100%: 1.11 [0.99 to 1.24]), but not with the inclusion of long sleep (aHR [95%CI] for 70 to 90%: 1.17 [1.07 to 1.28]; 90 to 100%: 1.21 [1.09 to 1.36]) or sleep efficiency (aHR for 70 to 90%: 1.16 [1.06 to 1.26]; 90 to 100%: 1.19 [1.06 to 1.33]; see *SI Appendix*, Table S6. The relationship of day light with all-cause mortality in Model 3 was not attenuated with the inclusion of short sleep (aHR for 50 to 70%: 0.91 [0.83 to 1.00]; 70 to 90%: 0.84 [0.76 to 0.93]; 90 to 100%: 0.84 [0.73 to 0.96]), long sleep (aHR for 50 to 70%: 0.90 [0.82 to 0.99]; 70 to 90%: 0.83 [0.74 to 0.91]; 90 to 100%: 0.81 [0.71 to 0.93]), or sleep efficiency (aHR for 50 to 70%: 0.90 [0.82 to 0.99]; 70 to 90%: 0.83 [0.75 to 0.92]; 90 to 100%: 0.82 [0.72 to 0.94]). Relationships of mean and minimum circadian amplitude with all-cause mortality were attenuated with the inclusion of short sleep (aHR for mean amplitude: 0.98 [0.94 to 1.01] per SD; minimum amplitude: 0.98 [0.95 to 1.01]), but not with the inclusion of long sleep or sleep efficiency (*SI Appendix*, Table S7). Relationships of maximum circadian amplitude, late circadian phase, and early circadian phase with all-cause mortality were not attenuated with the inclusion of short sleep, long sleep, or sleep efficiency.

## Discussion

Across 13 million hours of personal light exposure data from wrist-worn sensors in ~89,000 individuals, those exposed to brighter nights and darker days had a higher risk of all-cause mortality, and higher risk of mortality from cardiometabolic causes. Modeling the impact of light on the circadian system indicated that suppressed circadian amplitude and shifted circadian phase were associated with all-cause and cardiometabolic mortality, consistent with the known biological effects of light exposure on the circadian clock (5).

Exposure to more night light or less day light was associated with higher risk of all-cause mortality, and the relationship between light exposure and mortality risk was dose-dependent. Compared to individuals with low night light exposure (0 to 50th percentiles), individuals in the 70 to 90th percentiles of night light exposure had a 15 to 17% higher risk of all-cause mortality, while individuals in the 90 to 100th percentiles of night light exposure had a 21 to 34% higher risk of all-cause mortality. Compared to individuals with low day light exposure (0 to 50th percentiles), individuals in the 50 to 70th, 70 to 90th, and 90 to 100th percentiles of day light exposure had 10 to 16%, 16 to 26%, and 17 to 34% lower risks of all-cause mortality, respectively. Relationships of day and night light with mortality risk were robust to comprehensive adjustments for potentially confounding factors, including age, sex, ethnicity, photoperiod, socioeconomic advantage, physical activity, social activity, smoking, alcohol, urbanicity, shift work, and baseline cardiometabolic health. These findings are consistent with previous cohort studies demonstrating higher all-cause mortality risk for people living in areas with brighter nights (satellite-derived) (15), and for people with self-reported lower day light exposure (24).

The observed relationships of night light exposure with mortality risk may be explained by night light disrupting circadian rhythms, leading to adverse cardiometabolic outcomes. Brighter night light is linked to the development of metabolic syndrome, diabetes, and obesity (31, 46–50), and circadian disruption is strongly implicated in the development of cardiometabolic diseases including myocardial infarction, stroke, hypertension, and diabetes (32–35). Consistent with these findings, we demonstrated that exposure to brighter night light was associated with higher risk of mortality by cardiometabolic causes. Individuals in the 70 to 90th and 90 to 100th percentiles of night light exposure had 22 to 26% and 33 to 46% higher risks of cardiometabolic mortality, compared to those with dark nights (0 to 50th percentiles). In comparison, for other-cause mortality, individuals in the 70 to 90th and 90 to 100th percentiles of night light exposure had 13 to 15% and 17 to 30% higher risks, respectively. Analysis of mortality risk for light exposure across half-hour intervals showed a peak cardiometabolic risk between 02:30 and 03:00 (56 to 67% greater risk for brightest 10% vs. bottom 50%). This is consistent with evidence that circadian rhythms are most disrupted by light exposure across a short interval in the middle of the biological night (5, 51), and also consistent with our modeling results that link lower circadian amplitude and deviated circadian phase with higher cardiometabolic mortality risk.

Relationships of brighter day light exposure with lower mortality risk may be explained by the enhancing effects of day light on circadian rhythms (18–23), which protect against the negative health effects of circadian disruption. Enhancement of circadian rhythms by day light may explain why premature mortality and adverse cardiometabolic outcomes have been linked to day light exposure in previous cohort studies (24–27). Higher vitamin D levels due to higher sunlight exposure have been suggested as an explanation for relationships of day light exposure with mortality risk (24); however, causal evidence does not support this explanation for cardiometabolic mortality (52), and causal evidence for all-cause mortality is mixed (52, 53). Co-occurrence of physical activity with day light exposure is another plausible explanation for relationships of day light with mortality risk. We observed attenuated light-mortality relationships in models adjusted for objective physical activity, though brighter day light still predicted 10 to 17% lower mortality risks in these models, and in a dose-dependent manner. Personal day light exposure appears to be an independent predictor of mortality risk, and our modeling results support enhanced circadian amplitude as the potential mechanism linking brighter days with lower mortality risk.

Light synchronizes the timing of the brain’s central circadian pacemaker to the 24 h light/dark cycle, but mistimed light exposure can also cause suppression of circadian amplitude and shifted circadian phase (4, 5, 54). Using a validated computational model representing the dynamic response of the central circadian clock to light, we found that disrupted circadian rhythms predicted higher mortality risk. Each SD reduction in circadian amplitude was associated with 4 to 10% higher all-cause mortality risk and 7 to 13% higher cardiometabolic mortality risk. Individuals whose circadian phase minima occurred more than 1 h before the group average had a 16 to 30% higher risk of all-cause mortality, and a 27 to 43% higher risk of cardiometabolic mortality, and those with minima more than 1 h later than the group average had a 13 to 20% higher risk of all-cause mortality, consistent with the finding of higher mortality risk in both early and late sleepers (55). Together with the observed relationships of brighter nights and darker days with mortality risk, these findings support the notion that circadian disruption is a potential mechanism linking light exposure with mortality risk. This link could be explained by the role of circadian disruption in the initiation and progression of disease (1), by the disruption of circadian regulation in gene expression that correlates with premature mortality (56), or by a reduction in the central clock’s ability to organize peripheral rhythms (57).

This study investigated the relationship between personal light exposure and mortality risk in a large, well-characterized cohort, using wrist-worn light sensors. Previous large-scale studies have assessed satellite-derived outdoor light exposure, and self-reported day light exposure, finding associations with risk of premature mortality, and coronary heart disease (15, 16, 24, 26). Satellite data captures the outdoor environment only, and may not be an ideal proxy for an individual’s light exposure pattern, including indoor light levels (58). Furthermore, self-report data are subject to recall bias, and may not capture intraindividual variation in light exposure over time. Our analyses used data from personal sensors and therefore captured a range of lighting environments specific to each individual, at all clock times. Sensor data allowed for the inclusion of personal day and night light exposure together in mortality risk models, which is important given day light exposure can alter the sensitivity of the circadian system to light at night (21–23). Furthermore, personal data allowed us to model the effect of light exposure on each individual’s circadian clock, an approach that incorporates information about their light exposure history. This modeling approach supports the utility of light-derived circadian metrics for predicting human health outcomes (31), and expands upon research that derived circadian metrics from accelerometer data only (13, 14).

Previous studies have linked all-cause mortality with short, long, and inefficient sleep (40–43). Due to the bidirectional relationship between light–dark and sleep–wake patterns, it is possible that relationships of light exposure with mortality could be explained by disrupted or abnormal sleep–wake patterns. We found that including short sleep duration in our models attenuated the relationship of night light exposure with mortality, but not the relationship of day light with mortality risk. Long or fragmented sleep did not attenuate relationships of day or night light with mortality risk. Furthermore, sleep did not explain the relationships of early or late circadian phase with higher mortality risk, or the relationship of enhanced circadian amplitude (i.e., by bright day light exposure) with lower mortality risk. Taken together, these results indicate that short sleep partially explains the higher mortality risk observed in people with brighter night light exposure, but also that disrupted or enhanced circadian rhythms predict mortality risk independently from sleep disruption.

There are several limitations to this study. First, only 1 wk of light exposure was available for each participant. Light patterns, however, were stable across up to four repeated-measures collections in ~3,000 participants, indicating that 1 wk of data was a reasonable proxy for an individual’s typical light patterns (31, 59). Second, light recordings did not occur simultaneously with collection of several covariates that are subject to change over time. Third, the computational model was developed using studies of healthy younger adults (5, 60), and does not account for individual differences in physiology, including differences in light sensitivity (61), or possible age-related changes is sleep and circadian rhythms, such as advanced sleep timing relative to circadian phase (62). Fourth, the UK Biobank cohort was predominantly white ethnicity (97%), and it is therefore unclear whether these findings generalize to groups with different ancestry or sociocultural context. Finally, though we adjusted for many potential confounders, since this is a correlational study, it is possible that light exposure patterns and premature mortality are explained by other unmeasured factors.

These findings demonstrate the importance of maintaining a dark environment across the late night and early morning hours, when the central circadian pacemaker is most sensitive to light, and seeking bright light during the day to enhance circadian rhythms. Protection of lighting environments may be especially important in those at risk for both circadian disruption and mortality, such as in intensive care or aged-care settings (63, 64). Across the general population, avoiding night light and seeking day light may lead to reduction in disease burden, especially cardiometabolic diseases, and may increase longevity.

## Materials and Methods

### Light Exposure: Data Collection.

Approximately 502,000 UK Biobank participants aged between 40 and 69 y were recruited between 2006 and 2010 (65). From this cohort, 103,669 participants wore Axivity AX3 devices (Axivity, Newcastle upon Tyne, UK). Devices were distributed and returned by post. See *SI Appendix*, Table S8 and section S1 for additional detail on data collection protocol.

### Light Exposure: Devices.

Light and accelerometer data were logged at 100 Hz. Devices contained an APDS8007 silicon photodiode light sensor that responded to a spectral range similar to the human eye (peak sensitivity wavelength of 560 nm). We tested a sample of Axivity AX3 devices under reference lighting conditions, confirming an approximately linear response to illuminance between 0 and 5,500 lx, as reported previously (59). Raw device outputs were converted to approximate “lux” using the conversion formula specified by the device manual and were transformed in accordance with testing data (59).

### Light Exposure Profiles.

Light data were cleaned based on accelerometer data to ensure light recordings corresponded to when devices were on-wrist. Device nonwear was determined by GGIR, a validated package for estimating sleep–wake state from accelerometer data, as reported previously (45, 66, 67). Participants had a median (IQR) of 6.90 (5.95 to 6.96) days of light data remaining after exclusion of epochs coinciding with nonwear. Participants with no valid days detected by GGIR were excluded, due to nonwear or data corruption (8,004 of 103,669). Data from each 7-d light recording were grouped into a daily light profile, consisting of 48 half-hour intervals representing all clock times across 24 h (e.g., all light data between 00:00 and 00:30 across 7 d). We excluded participants with low-quality light data reflecting device malfunction, or insufficient data in any of the 48 half-hour clock time intervals. There were 210 possible minutes of cumulative light data across 7 d within each half-hour clock time interval. Participants with <60 out of 210 min of data in any of their half-hour clock time intervals were excluded (6,761 of 95,665 participants excluded). Daily light profiles representing all 24-h clock times remained in 88,905 participants (see *SI Appendix*, section S1 for additional detail).

### Cause-Specific Mortality.

Cardiometabolic mortality was defined as any cause of death corresponding to ICD-10 diseases of the circulatory system, or endocrine and metabolic diseases. Predominant circulatory causes of death were ischemic heart disease (I20-I25), cerebrovascular diseases (I60-I69), other heart disease (I30-I52), diseases of the arteries, arterioles, and capillaries (I70-I79), and hypertensive diseases (I10-I15). Predominant endocrine and metabolic causes of death were diabetes mellitus (E10-E14), metabolic disorders (E70-E90), and obesity (E65-E68).

### Covariates.

Baseline covariates were collected between 2006 and 2010, including self-reported ethnic background, employment status, yearly household income, education, Townsend Deprivation Index (average deprivation of participants’ residential area), smoking status (previous/never/current), alcohol consumption (days per week), urbanicity (residential area >10,000 population), number of weekly social activities, frequency of social visits, and shift work status (job involved any shift work). Photoperiod was calculated as the interval from sunrise to sunset at 53.4808°N, 2.2426°W (Manchester, UK) on the date of light recording. Physical activity was included as the acceleration average across each weekly recording, as derived in previous work (68). Cardiometabolic risk factors included BMI, hypertension, cholesterol ratio (total cholesterol/HDL), diabetes diagnosis, and history of vascular conditions. Sleep duration, sleep efficiency, and midsleep were estimated using GGIR, a validated package for estimating sleep–wake state from accelerometer data, as reported previously (45, 66, 67). See *SI Appendix*, Tables S9 and S10 for detailed descriptions of covariates.

### Statistical Analysis.

Hazard ratios for all-cause and cause-specific mortality were estimated using Cox proportional hazards models and competing-risks proportional subhazards models (37). Time since light recording was used as the timescale and all models were adjusted for participant age. Light data were split into four percentile groupings: 0 to 50% (referent group), 50 to 70%, 70 to 90%, and 90 to 100%. The 0 to 50th percentiles were grouped due to minimal variability in their average illuminance at night, and due to the skewed nature of light data. The 0 to 50th percentile groups were hypothesized to have the lowest risk of mortality for night light, and the highest risk of mortality for day light. See *SI Appendix*, section S1 for further detail on model implementation and covariate inclusion in Models 1 to 3.

### Circadian Rhythm Modeling.

Circadian phase and amplitude were modeled from each participant’s approximately 7-d light recording. A detailed description of model equations and implementation is provided in *SI Appendix*, section S1. Amplitude was calculated at each epoch of the light recording. Mean, minimum, and maximum amplitudes were calculated over this time series for each participant. Phase (predicted time of core body temperature minimum) was calculated for each ~24 h cycle, and mean and SD of phase were calculated from this set of phase values for each participant.

Amplitude metrics and phase variability were z-scored for inclusion as continuous predictors of all-cause, cardiometabolic, and noncardiometabolic mortality in Cox proportional hazards and subhazards models. Mean phase was split into quintiles to account for the circular nature of the data. The 40 to 60th percentile group was centered at participants’ circular mean phase (03:50) and was used as a referent group in Cox models.

### Ethics.

The UK Biobank has ethical approval from the North West Multi-centre Research Ethics Committee (https://www.ukbiobank.ac.uk/learn-more-about-uk-biobank/about-us/ethics).

## Supplementary Material

Appendix 01 (PDF)

## Data Availability

Previously published data were used for this work. All data are available on the UK Biobank website: https://biobank.ndph.ox.ac.uk/showcase/search.cgi (65). Links to all UK Biobank data fields used in this work are available in *SI Appendix*, Table S9.
