# Supplementary material for: Brighter nights and darker days predict higher mortality risk: A prospective analysis of personal light exposure in >88,000 individuals
Source: Proc Natl Acad Sci U S A. 2024 Oct 15;121(43):e2405924121. doi: 10.1073/pnas.2405924121 (PMC11513964; doi:10.1073/pnas.2405924121)
Supplement: Supplementary file 1 — Appendix 01 (PDF) [file pnas.2405924121.sapp.pdf]

Supporting Information: Brighter nights and darker days predict higher mortality risk: A prospective analysis of personal light exposure in >88,000 individuals

Daniel P. Windred[1,2], Angus C. Burns[3-6], Jacqueline M. Lane[3-6], Patrick Olivier[7], Martin K. Rutter[8,9], Richa Saxena[3,4,6,10], Andrew J. K. Phillips[1,2]*, & Sean W. Cain[1,2]*

Affiliation / Institution:

[1]Flinders Health and Medical Research Institute (Sleep Health), Flinders University, Bedford Park, SA, Australia

[2]School of Psychological Sciences, Faculty of Medicine, Nursing and Health Sciences, Monash University, Melbourne, VIC, Australia

[3]Division of Sleep and Circadian Disorders, Brigham and Women's Hospital, Boston, MA, USA

[4]Division of Sleep Medicine, Harvard Medical School, Boston, MA, USA

[5]Program in Medical and Population Genetics, Broad Institute, Cambridge, MA, USA

[6]Center for Genomic Medicine, Massachusetts General Hospital, Boston, MA, USA

[7]Action Lab, Department of Human-Centred Computing, Faculty of Information Technology, Monash University, Melbourne, VIC, Australia

[8]Centre for Biological Timing, Division of Endocrinology, Diabetes & Gastroenterology, School of Medical Sciences, Faculty of Biology, Medicine and Health, Manchester Academic Health Science Centre, University of Manchester, Manchester, UK

[9]Diabetes, Endocrinology and Metabolism Centre, NIHR Manchester Biomedical Research Centre, Manchester University NHS Foundation Trust, Manchester, UK

[10]Department of Anesthesia, Critical Care and Pain Medicine, Massachusetts General Hospital and Harvard Medical School, Boston, MA, USA

*Authors contributed equally to this manuscript.

Corresponding Authors: Sean W. Cain (sean.cain@flinders.edu.au); Daniel P. Windred (daniel.windred@flinders.edu.au)

Keywords: light sensor; light at night; circadian disruption; longevity; cardiometabolic

Classification: Biological Sciences / Medical Sciences

Table of Contents

Figure S1. Approximated mean illuminance (lux) by half-hour clock time intervals across 88,905 participants... 3

Section S1. Supporting Materials and Methods ... 4

Table S1. Descriptives statistics for participants grouped according to light exposure percentiles, and split by day and night light ... 7

Table S2. Day light, night light, and mortality risk, adjusted for baseline cardiometabolic health and shift work 8

Figure S2. Risk of all-cause, cardiometabolic, and other-cause mortality for light exposures across 24 h (Model 2) ... 9

Table S3. Modeled circadian phase, amplitude, and mortality risk, adjusted for baseline cardiometabolic health and shift work ... 10

Table S4. Percentage and number of deaths within quintiles of modeled circadian variables ... 11

Table S5. Relationships of modeled circadian phase with midsleep timing ... 12

Table S6. Day light, night light and mortality risk, adjusted for sleep duration and efficiency ... 13

Table S7. Modeled circadian phase, amplitude, and mortality risk, adjusted for sleep duration and efficiency 14

Table S8. UK Biobank protocol documents ... 15

Table S9. UK Biobank variables ... 16

Table S10. Covariates included in statistical models, as derived from UK Biobank variables ... 18

Table S11. STROBE Checklist ... 19

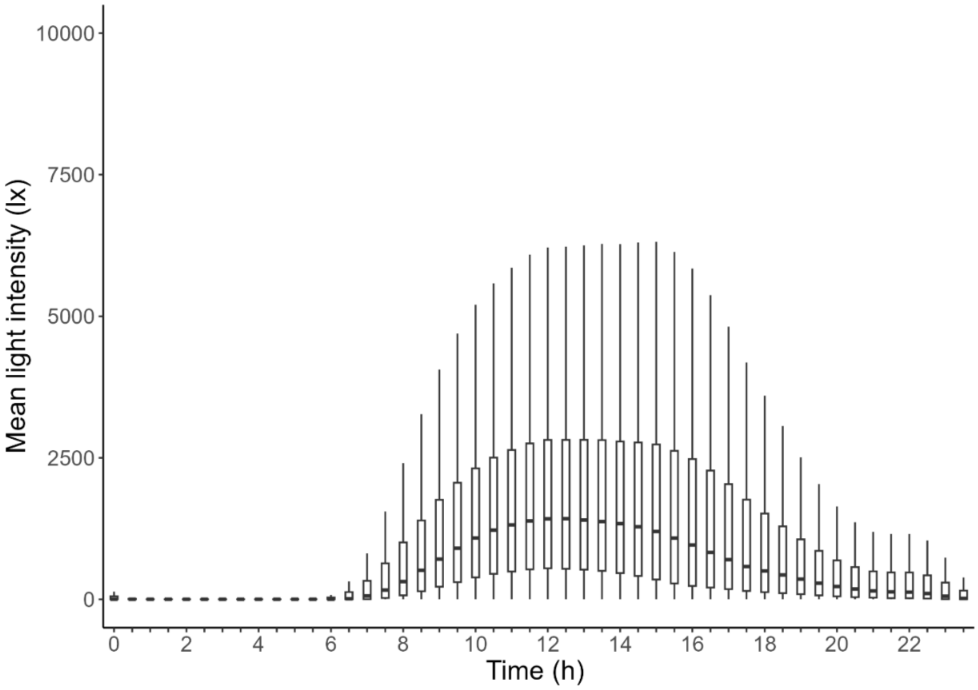

Figure S1. Approximated mean illuminance (lux) by half-hour clock time intervals across 88,905 participants

Section S1. Supporting Materials and Methods

Protocol

The initial cohort of approximately 502,000 participants underwent assessment at one of twenty-two assessment centres across the UK, located to capture the range of ethnic, socio-economic, and urban-rural distributions across the UK population, between 2006-2010. Invitations to participate in activity/light tracking were sent to 236,519 individuals from the initial cohort via post, and 103,669 agreed to participate, between 2013-2016. Documentation relating to consent and recruitment are available on the UK Biobank website, and relevant links are provided in Table S10.

Light Data Cleaning

Raw data from Axivity AX3 devices were converted to device lux based on the device manual, where device lux = 10^(device output current/341). Data were downsampled to 1Hz, and the median of all light data within 10-minute rolling windows, stepped in 2-minute increments, was extracted.

Night Light and Day Light Factors

Night light (00:30-06:00) and day light (07:30-20:30) groupings were extracted using factor analysis, based on factor loading ≥0.5 (varimax rotation, cumulative proportion variance explained = 0.56) (1). Day and night light factors were internally consistent (Cronbach's α=.98 and α=.93, respectively), and exhibited a weak positive correlation (r_s=0.10, p<.0001).

Circadian Rhythm Modeling

Light data was input to a dynamic stimulus processer that represents transduction and transmission of light from the environment to the central pacemaker, via photoreceptors and the retino-hypothalamic tract (Process L). Output from Process L was input to a limit cycle oscillator, which represents the central pacemaker (Process P).

Process L was modeled by:

$$\dot{n} = C(\alpha(1-n) - \beta n)$$

$$\alpha = \alpha_0 \left(\frac{I}{I_0}\right)^p$$

where n represents the fraction of saturated photoreceptor elements, and I represents light intensity.

The central pacemaker (Process P) was modeled by:

$$\dot{x} = \frac{\pi}{12}\left(x_c + \hat{B}(1-0.4x)(1-0.4x_c)\right)$$

$$\dot{x}_c = \frac{\pi}{12}\left(\mu\left(x_c - \frac{4x_c^3}{3}\right) - x\left(\left(\frac{24}{0.99729\tau_x}\right)^2 + k\hat{B}(1-0.4x)(1-0.4x_c)\right)\right)$$

$$\hat{B} = G(1-n)\alpha$$

where x and x_c represent the state of the central circadian pacemaker, and $\hat{B}$ represents the drive on the pacemaker from the transmission of light information from Process L.

Light intensity (I), photoreceptor state (n), and circadian pacemaker state (x, x_c) are all functions of time, defined at all epochs of each participant's light data recording.

Fixed parameters:

$C = 60$

$\beta = 0.013\, min^{-1}$

$\alpha_0 = 0.16\, min^{-1}$

$I_0 = 9500$

$p = 0.6$

$\mu = 0.23$

$G = 19.875$

$\tau_x = 24.2$

$k = 0.55$

Phase was calculated for each approximate 24 h interval, as clock time at the minimum value of x plus a reference value of 0.8h. Phase was extracted for all available days of light data, and mean and standard deviation were calculated across these days for each participant.

Amplitude was calculated by:

$$A = \sqrt{x^2 + x_c^2}$$

for pairs of x and x_c at all epochs. Mean, minimum, and maximum amplitude were calculated from amplitude across all epochs.

Initial conditions for Process P were defined according to each participant's weekly average midsleep time, commencing on the limit cycle. To minimize the transient effects of initial conditions on model outputs, we replicated each participant's data to 35 days in length (e.g., 7 days of light data were replicated 5 times, concatenated, and input to the model as a 35 day interval). Phase and amplitude were then calculated based on the last N days of the replicated times series, where N was number of days of data available for each participant.

Since light data contained missing epochs due to non wear, we also imputed any length of missing light data ≤120 min using Kalman imputation with the 'imputeTS' package in R. We then extracted the longest continuous interval of light data for each participant containing whole days only, excluding all participants with <3 continuous days of light data (95,068 remaining after exclusion). Participants with daily light profiles that did not capture light exposure across 24 h were also excluded, as described above and in the main text. There were 85,003 participants with light data suitable for input to the circadian rhythm model. There were 6 days of data remaining in 65.8% of participants, 5 days in 21.2%, 4 days in 6.9%, and 3 days in 6.0%. A median (IQR) of 0.57 (0.80) h of data were imputed 73% of participants, and no imputation was required in 27% of participants.

Sleep Estimation

Sleep was derived using GGIR (2, 3), a validated open-source R package for estimating sleep-wake state from accelerometer data. This package was implemented as reported previously (4). Sleep duration (M ± SD = 6.79 ± 1.05 h) was calculated as the total duration of sustained inactivity between nightly 'sleeponset' and 'wakeup' times estimated by GGIR, where sustained inactivity represented <5° of deviation of the accelerometer from its z-axis for >5 min. The mean of sleep duration was calculated using all available nights in each participant, after excluding all participants with <3 nights of sleep data in accordance with GGIR output.

Cox Proportional Hazards Implementation

Cox proportional hazards models were implemented using the 'survival' package in R (version 4.1.0), and competing risks models using the 'crrSC' package. Survival curves were adjusted using the 'survminer' package implementing a 'marginal' approach, which balances each subgroup across all covariates. Contrasts were set using the default 'contr.treatment' setting in R for all categorical variables. This method defines a referent category to which all other categories are compared (e.g., 0-50% light exposure category defined as referent, 50-70%, 70-90%, 90-100% exposure categories as comparison groups).

An example of implemented model syntax for cox proportional hazards models is as follows:

coxph(Surv(years, death) ~ light_night + light_day + age + sex + ethnicity, data=data)

where *years* represents time between light recording and either death or censoring, and *death* is a binary marker of participant survival.

An example of model syntax for proportional sub-hazards models is as follows:

crrs(ftime = years, fstatus = death_type, cov1 = covs, failcode = n, strata = strata_covs, ctype = 1)

where *years* represents time between light recording and either death or censoring, *death_type* is a numerical representation of cause of death (0 = living, 1 = cardiometabolic, 2 = non-cardiometabolic), *covs* is data structure containing required covariates (e.g., age, sex, ethnicity), *n* represents the cause of death defined as the competing hazard in each model (e.g., n = 1 to test the competing hazard of cardiometabolic mortality), and *strata_covs* represents covariates included as 'strata' variables.

The assumption of proportional hazards was assessed by inspecting the plotted temporal stability of regression coefficients associated with each covariate, using the 'cox.zph' function. Multi-collinearity was assessed by calculating the Variance Inflation Factor (VIF) for all model covariates using the 'ols_vif_tol' function. Age, physical activity, diabetes status, vascular diagnoses, BMI, hypertension, and cholesterol ratio were included in all models as 'strata' variables, accounting for group-wise differences in baseline mortality risk.

Variables known to influence mortality and light exposure were chosen as model covariates. Age, sex, ethnicity, and photoperiod were included in Model 1, as characteristics that may only exhibit a unidirectional causal relationship with both mortality and light exposure. Additional covariates in Models 2 and 3 were selected due to their possible confounding effect on the mortality/light exposure relationship. These covariates were also potential mediators of light-mortality relationships, in contrast with age, sex, and ethnicity which were not.

References

1. A. C. Burns *et al.*, Day and night light exposure are associated with psychiatric disorders: an objective light study in> 85,000 people. *Nat. Ment. Health* 1, 853-862 (2023).
2. J. H. Migueles, A. V. Rowlands, F. Huber, S. Sabia, V. T. van Hees, GGIR: a research community–driven open source R package for generating physical activity and sleep outcomes from multi-day raw accelerometer data. *J. Meas. Phys. Behav.* 2, 188-196 (2019).
3. V. T. van Hees *et al.*, Estimating sleep parameters using an accelerometer without sleep diary. *Sci. Rep.* 8, 1-11 (2018).
4. D. P. Windred *et al.*, Objective assessment of sleep regularity in 60 000 UK Biobank participants using an open-source package. *Sleep* 44, zsab254 (2021).

Table S1. Descriptives statistics for participants grouped according to light exposure percentiles, and split by day and night light

	**Night light exposure percentiles**				**Day light exposure percentiles**			
	0-50%	50-70%	70-90%	90-100%	0-50%	50-70%	70-90%	90-100%
Light exposure (lux)								
Median (IQR)	0.6 (0.5-0.8)	2.5 (1.6-3.9)	16.4 (10.1-27.2)	105 (69.4-191)	426 (221-675)	1320 (1150-1520)	2310 (2000-2680)	3810 (3450-4350)
Range	<1.2	1.2 - 6.3	6.3 - 48.3	>48.3	4.3 - 991	991 - 1750	1750 - 3140	>3140
Photoperiod (range: 7.5-17.0 h)								
M±SD	12.2 ± 3.0	12.9 ± 3.2	12.8 ± 3.3	12.6 ± 3.7	10.9 ± 2.9	12.9 ± 2.9	14.5 ± 2.3	15.7 ± 1.5
Age								
M ± SD	62.8 ± 7.9	61.8 ± 7.9	62.0 ± 7.8	62.4 ± 7.7	62.1 ± 8.0	62.4 ± 7.8	62.6 ± 7.7	63.5 ± 7.4
Range	43.5 - 78.9	43.5 - 79.0	43.7 - 78.8	43.8 - 78.4	43.6 - 79.0	43.6 - 78.7	43.5 - 78.8	43.5 - 78.5
Sex (% male, N)	41.3 (18353)	45.0 (7998)	44.7 (7949)	45.2 (4022)	42.2 (18774)	42.1 (7478)	43.4 (7719)	48.9 (4351)
Ethnicity (% white, N)	97.7 (43296)	96.7 (17133)	96.2 (17033)	95.6 (8462)	96.3 (42669)	97.0 (17190)	97.7 (17315)	98.7 (8750)
Employment status (% employed, N)	59.7 (26360)	64.5 (11388)	65.0 (11474)	63.7 (5622)	63.9 (28201)	61.8 (10903)	60.7 (10720)	56.9 (5020)
Income								
% <£18k, N	12.9 (5679)	13.1 (2307)	12.9 (2278)	14.0 (1238)	13.5 (5959)	12.9 (2283)	12.6 (2221)	11.7 (1039)
% £18-29.9k, N	22.1 (9753)	21.4 (3779)	21.2 (3747)	21.8 (1924)	21.6 (9537)	22.0 (3884)	21.6 (3815)	22.2 (1967)
% £30-51.9k, N	25.9 (11452)	26.3 (4637)	26.0 (4592)	25.3 (2231)	25.9 (11431)	25.4 (4489)	26.4 (4662)	26.3 (2330)
% £52-100k, N	22.7 (10016)	23.1 (4073)	23.4 (4129)	22.2 (1957)	22.8 (10058)	22.8 (4017)	23.1 (4079)	22.8 (2021)
% >£100k, N	6.27 (2767)	6.90 (1219)	7.14 (1261)	7.30 (644)	6.61 (2917)	6.50 (1147)	6.90 (1220)	6.85 (607)
Education								
% other, N	48.1 (21144)	48.4 (8529)	48.6 (8547)	48.9 (4300)	47.8 (21013)	48.7 (8552)	48.7 (8577)	49.6 (4378)
% university/college, N	43.4 (19105)	43.4 (7639)	43.5 (7657)	42.9 (3775)	44.0 (19367)	43.2 (7596)	43.0 (7566)	41.4 (3647)
Townsend Deprivation Index								
M ± SD	-1.9 ± 2.7	-1.7 ± 2.8	-1.6 ± 2.9	-1.4 ± 3.0	-1.6 ± 2.9	-1.8 ± 2.8	-1.9 ± 2.7	-2.3 ± 2.4
Range	-6.3 - 10.5	-6.3 - 9.9	-6.3 - 10.0	-6.3 - 9.9	-6.3 - 10.5	-6.3 - 9.9	-6.3 - 9.9	-6.3 - 8.9
Smoking								
% previous, N	34.8 (15409)	36.1 (6391)	37.9 (6725)	38.8 (3439)	35.2 (15579)	36.1 (6396)	36.9 (6539)	38.9 (3450)
% current, N	5.44 (2411)	7.27 (1288)	8.18 (1451)	10.2 (907)	7.06 (3130)	7.04 (1247)	6.58 (1167)	5.78 (513)
Alcohol (M ± SD, days per week)	3.97 ± 2.48	2.98 ± 2.50	2.99 ± 2.51	2.94 ± 2.55	2.87 ± 2.48	2.98 ± 2.50	3.07 ± 2.51	3.29 ± 2.53
Urbanicity (% >10,000 population, N)	83.2 (36620)	83.7 (14741)	85.6 (15075)	86.5 (7598)	85.5 (37603)	84.1 (14799)	83.1 (14628)	79.4 (7004)
Physical activity								
M ± SD	27.9 ± 7.9	28.6 ± 8.3	28.5 ± 8.2	27.8 ± 8.3	27.3 ± 7.9	28.2 ± 8.0	29.0 ± 8.1	30.6 ± 8.5
Range	4.8 - 69.2	6.5 - 69.3	5.9 - 69.2	5.1 - 69.4	5.1 - 69.3	4.9 - 67.9	6.5 - 69.4	4.8 - 67.4
Social activities (% >1 weekly, N)	72.7 (32250)	72.9 (12926)	73.3 (13015)	71.8 (6364)	72.1 (31973)	72.5 (12856)	73.6 (13063)	75.0 (6663)
Social visits								
% never, N	1.1 (475)	1.2 (218)	1.3 (227)	1.6 (140)	1.4 (611)	1.1 (201)	1.0 (178)	0.8 (70)
% every few months, N	6.9 (3027)	7.1 (1261)	7.0 (1242)	7.0 (617)	7.3 (3224)	6.7 (1190)	6.5 (1152)	6.6 (581)
% monthly, N	15.1 (6659)	14.5 (2561)	14.0 (2475)	15.0 (1323)	15.0 (6621)	14.9 (2633)	14.3 (2539)	13.8 (1225)
% weekly, N	36.5 (16126)	36.2 (6402)	36.3 (6410)	35.5 (3143)	36.2 (16017)	36.1 (6384)	36.3 (6428)	36.7 (3252)
% 2-4 times per week, N	30.7 (13572)	30.5 (5392)	30.9 (5471)	30.1 (2667)	30.1 (13314)	31.0 (5474)	31.4 (5562)	31.1 (2752)
% almost daily, N	9.7 (4309)	10.3 (1823)	10.4 (1837)	10.7 (945)	9.8 (4347)	10.0 (1763)	10.3 (1829)	11.0 (975)

Table S2. Day light, night light, and mortality risk, adjusted for baseline cardiometabolic health and shift work

		**Light exposure percentile**	**Cases % (N)**	**HR [95% CI]**	**p-value**
Model 3 + vascular conditions	Night	0-50% (ref.)	4.06 (1734)	-	<0.0001
N = 85,494		50-70%	3.88 (664)	1.01 [0.92-1.11]	0.83
		70-90%	4.46 (763)	1.14 [1.05-1.25][a]	0.0025
		90-100%	4.95 (423)	1.20 [1.08-1.34][a]	0.0011
	Day	0-50% (ref.)	4.31 (1843)	-	<0.0001
		50-70%	4.11 (702)	0.91 [0.83-0.99][a]	0.035
		70-90%	3.94 (674)	0.84 [0.76-0.93][a]	0.00073
		90-100%	4.27 (365)	0.83 [0.73-0.95][a]	0.0065
Model 3 + diabetes	Night	0-50% (ref.)	4.05 (1731)	-	<0.0001
N = 85,451		50-70%	3.87 (662)	1.01 [0.92-1.10]	0.91
		70-90%	4.46 (762)	1.14 [1.05-1.25][a]	0.0025
		90-100%	4.93 (421)	1.19 [1.07-1.33][a]	0.0015
	Day	0-50% (ref.)	4.30 (1838)	-	<0.0001
		50-70%	4.10 (700)	0.90 [0.82-0.99][a]	0.028
		70-90%	3.94 (673)	0.84 [0.76-0.93][a]	0.00085
		90-100%	4.27 (365)	0.83 [0.73-0.95][a]	0.0065
Model 3 + BMI >30	Night	0-50% (ref.)	4.06 (1732)	-	<0.0001
N = 85,396		50-70%	3.88 (662)	1.00 [0.91-1.09]	0.98
		70-90%	4.46 (762)	1.14 [1.04-1.24][a]	0.0041
		90-100%	4.94 (422)	1.18 [1.06-1.32][a]	0.0027
	Day	0-50% (ref.)	4.30 (1837)	-	<0.0001
		50-70%	4.11 (702)	0.90 [0.83-0.99][a]	0.03
		70-90%	3.95 (675)	0.84 [0.76-0.93][a]	6e-04
		90-100%	4.26 (364)	0.82 [0.72-0.94][a]	0.0031
Model 3 + hypertension	Night	0-50% (ref.)	4.06 (1654)	-	<0.0001
N = 81,398		50-70%	3.83 (624)	1.01 [0.92-1.10]	0.91
		70-90%	4.37 (711)	1.13 [1.04-1.24][a]	0.0055
		90-100%	4.94 (402)	1.21 [1.08-1.36][a]	0.00069
	Day	0-50% (ref.)	4.27 (1739)	-	<0.0001
		50-70%	4.05 (659)	0.90 [0.82-0.99][a]	0.03
		70-90%	3.94 (642)	0.85 [0.76-0.94][a]	0.0016
		90-100%	4.31 (351)	0.84 [0.74-0.96][a]	0.012
Model 3 + high cholesterol ratio	Night	0-50% (ref.)	4.06 (1504)	-	<0.0001
N = 74,028		50-70%	3.88 (574)	1.00 [0.90-1.10]	0.95
		70-90%	4.40 (652)	1.13 [1.03-1.25][a]	0.0079
		90-100%	4.90 (363)	1.19 [1.06-1.33][a]	0.0041
	Day	0-50% (ref.)	4.30 (1591)	-	<0.0001
		50-70%	4.11 (608)	0.91 [0.83-1.01]	0.067
		70-90%	3.92 (580)	0.84 [0.75-0.93][a]	0.0013
		90-100%	4.24 (314)	0.83 [0.72-0.95][a]	0.0079
Model 3, excluding shift workers	Night	0-50% (ref.)	4.21 (1658)	-	<0.0001
N = 78,682		50-70%	4.04 (636)	1.03 [0.94-1.13]	0.57
		70-90%	4.53 (713)	1.15 [1.05-1.26][a]	0.0021
		90-100%	5.11 (402)	1.23 [1.10-1.38][a]	0.00022
	Day	0-50% (ref.)	4.44 (1745)	-	<0.0001
		50-70%	4.23 (666)	0.91 [0.83-1.00]	0.052
		70-90%	4.09 (644)	0.85 [0.77-0.94][a]	0.002
		90-100%	4.50 (354)	0.84 [0.74-0.96][a]	0.011

[a]p<.05. Hazard ratios represent risks of all-cause mortality across light exposure percentile groups, compared to a reference light exposure group, for day and night light. Model 3 was adjusted for age, sex, ethnicity, photoperiod, employment status, education, income, deprivation, physical activity, smoking status, alcohol consumption, urbanicity, and social activity.

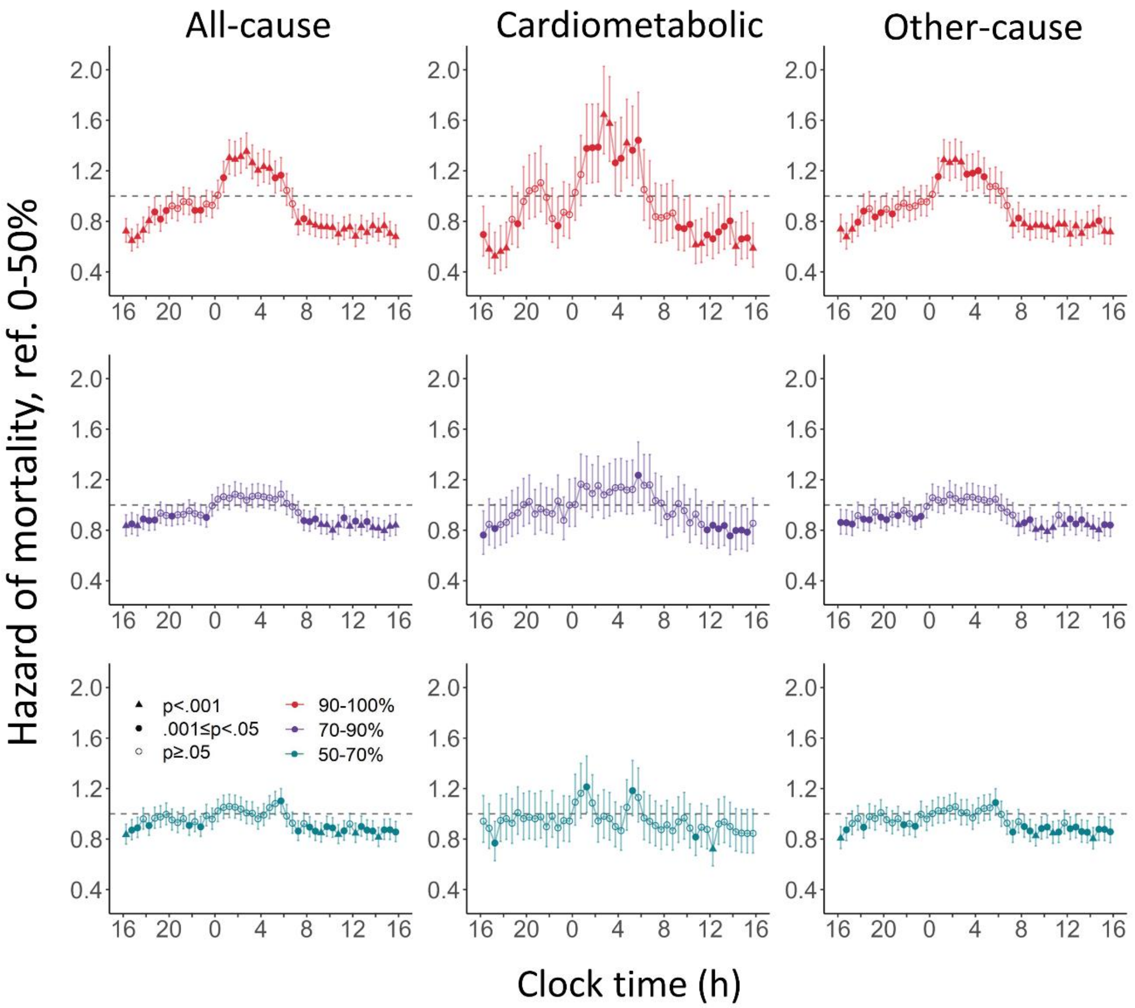

Figure S2. Risk of all-cause, cardiometabolic, and other-cause mortality for light exposures across 24 h (Model 2). Data are hazard ratios [95%CI]. Separate models were implemented for each half-hour clock time interval, with each model including 50-70%, 70-90%, and 90-100% light percentile groups referenced against 0-50%. Bonferroni correction for multiple comparisons required p<.001 for statistical significance.

Table S3. Modeled circadian phase, amplitude, and mortality risk, adjusted for baseline cardiometabolic health and shift work

		HR [95%CI]	p-value
Model 3 + vascular conditions	Mean Amplitude	0.96 [0.92-0.99][a]	0.0083
	Min. Amplitude	0.96 [0.93-0.99][a]	0.0054
	Max. Amplitude	0.94 [0.91-0.98][a]	0.0013
	Phase Variability	1.01 [0.98-1.04]	0.5
	Mean Phase		
	0-20%	1.16 [1.04-1.29][a]	0.0061
	20-40%	1.11 [0.99-1.24]	0.066
	40-60% (ref.)	-	-
	60-80%	1.08 [0.96-1.21]	0.18
	80-100%	1.12 [1.00-1.26][a]	0.045
Model 3 + diabetes	Mean Amplitude	0.96 [0.92-0.99][a]	0.0096
	Min. Amplitude	0.96 [0.93-0.99][a]	0.0075
	Max. Amplitude	0.94 [0.91-0.98][a]	0.0013
	Phase Variability	1.01 [0.98-1.03]	0.54
	Mean Phase		
	0-20%	1.15 [1.04-1.29][a]	0.009
	20-40%	1.11 [0.99-1.24]	0.076
	40-60% (ref.)	-	-
	60-80%	1.08 [0.96-1.21]	0.19
	80-100%	1.12 [1.00-1.25]	0.055
Model 3 + BMI >30	Mean Amplitude	0.96 [0.93-0.99][a]	0.015
	Min. Amplitude	0.96 [0.93-0.99][a]	0.018
	Max. Amplitude	0.94 [0.90-0.97][a]	0.00095
	Phase Variability	1.01 [0.98-1.03]	0.64
	Mean Phase		
	0-20%	1.16 [1.04-1.29][a]	0.008
	20-40%	1.11 [0.99-1.24]	0.062
	40-60% (ref.)	-	-
	60-80%	1.08 [0.96-1.21]	0.2
	80-100%	1.12 [1.00-1.26][a]	0.045
Model 3 + hypertension	Mean Amplitude	0.96 [0.93-1.00][a]	0.033
	Min. Amplitude	0.96 [0.93-0.99][a]	0.009
	Max. Amplitude	0.95 [0.91-0.98][a]	0.0043
	Phase Variability	1.01 [0.99-1.04]	0.36
	Mean Phase		
	0-20%	1.17 [1.04-1.30][a]	0.0065
	20-40%	1.11 [0.99-1.25]	0.069
	40-60% (ref.)	-	-
	60-80%	1.07 [0.95-1.20]	0.24
	80-100%	1.14 [1.01-1.28][a]	0.029
Model 3 + high cholesterol ratio	Mean Amplitude	0.96 [0.92-0.99][a]	0.015
	Min. Amplitude	0.96 [0.93-1.00][a]	0.036
	Max. Amplitude	0.94 [0.90-0.97][a]	0.0012
	Phase Variability	1.01 [0.98-1.04]	0.4
	Mean Phase		
	0-20%	1.16 [1.03-1.30][a]	0.012
	20-40%	1.11 [0.98-1.25]	0.092
	40-60% (ref.)	-	-
	60-80%	1.08 [0.96-1.22]	0.2
	80-100%	1.11 [0.98-1.26]	0.09
Model 3, excluding shift workers	Mean Amplitude	0.96 [0.93-0.99][a]	0.022
	Min. Amplitude	0.96 [0.93-0.99][a]	0.0045
	Max. Amplitude	0.95 [0.91-0.98][a]	0.0041
	Phase Variability	1.01 [0.98-1.04]	0.46
	Mean Phase		
	0-20%	1.16 [1.04-1.30][a]	0.0073
	20-40%	1.11 [0.99-1.25]	0.069
	40-60% (ref.)	-	-
	60-80%	1.07 [0.95-1.20]	0.26
	80-100%	1.12 [1.00-1.26]	0.056

[a]p<.05. Hazard ratios represent difference in mortality hazard per standard deviation increase in each circadian metric for mean, min., and max. amplitude, and phase variability. Hazard ratios for mean phase represent hazard of each percentile group relative to the 40-60% reference group, centered at the population mean phase (03:50). Model 3 was adjusted for age, sex, ethnicity, photoperiod, employment status, education, income, deprivation, physical activity, smoking status, alcohol consumption, urbanicity, and social activity.

Table S4. Percentage and number of deaths within quintiles of modeled circadian variables

		Deaths in circadian variable quintiles % (N)				
**Circadian variable**		Q1	Q2	Q3	Q4	Q5
Mean Amplitude	All-cause	4.63 (793)	4.09 (701)	3.89 (667)	4.25 (729)	4.13 (707)
	Non-cardiometabolic	1.16 (198)	0.76 (131)	0.77 (132)	0.97 (166)	0.82 (141)
	Cardiometabolic	3.43 (587)	3.26 (559)	3.12 (534)	3.26 (559)	3.29 (563)
Min Amplitude	All-cause	4.61 (789)	3.92 (672)	3.81 (653)	4.03 (690)	4.63 (793)
	Non-cardiometabolic	1.04 (179)	0.86 (147)	0.77 (132)	0.75 (128)	1.06 (182)
	Cardiometabolic	3.51 (602)	3.02 (518)	3.00 (514)	3.27 (561)	3.54 (607)
Max Amplitude	All-cause	4.56 (782)	4.17 (714)	4.09 (701)	4.38 (751)	3.79 (649)
	Non-cardiometabolic	1.04 (179)	0.83 (142)	0.89 (152)	0.97 (167)	0.75 (128)
	Cardiometabolic	3.47 (594)	3.31 (568)	3.15 (540)	3.39 (581)	3.03 (519)
Average Phase	All-cause	4.98 (853)	4.05 (693)	3.56 (611)	3.92 (671)	4.49 (769)
	Non-cardiometabolic	1.12 (192)	0.89 (152)	0.71 (121)	0.88 (150)	0.89 (153)
	Cardiometabolic	3.84 (657)	3.11 (533)	2.83 (485)	3.03 (519)	3.55 (608)
Phase Variability	All-cause	4.45 (763)	4.46 (764)	4.28 (733)	3.86 (662)	3.94 (675)
	Non-cardiometabolic	0.95 (162)	0.89 (153)	0.96 (164)	0.77 (132)	0.92 (157)
	Cardiometabolic	3.47 (594)	3.53 (605)	3.30 (565)	3.08 (527)	2.98 (511)

Table S5. Relationships of modeled circadian phase with midsleep timing

		Estimate	Std. Error	p-value
Midsleep (one week)	Mean Phase			
F (df) = 687.8 (4, 83,696)	0-20%	-0.365[a]	0.011	<.0001
	20-40%	-0.024[a]	0.011	0.036
	40-60% (ref.)	-	-	
	60-80%	0.033[a]	0.011	0.0037
	80-100%	0.211[a]	0.011	<.0001
Midsleep (weekends)[b]	Mean Phase			
F (df) = 358.4 (4, 69,683)	0-20%	-0.336[a]	0.015	<.0001
	20-40%	-0.021	0.015	0.17
	40-60% (ref.)	-	-	
	60-80%	0.043[a]	0.015	0.0043
	80-100%	0.227[a]	0.015	<.0001
Midsleep (weekdays)[b]	Mean Phase			
F (df) = 623.5 (4, 83,571)	0-20%	-0.375[a]	0.012	<.0001
	20-40%	-0.027[a]	0.012	0.022
	40-60% (ref.)	-	-	
	60-80%	0.030[a]	0.012	0.012
	80-100%	0.205[a]	0.012	<.0001

[a]p<.05. [b]Midsleep on weekends and weekdays were calculated for individuals with at least two nights of sleep data. Data are estimated coefficients for single linear regression models predicting midsleep timing from modeled circadian phase.

Table S6. Day light, night light and mortality risk, adjusted for sleep duration and efficiency

		Light intensity percentile	All-cause mortality HR [95%CI]	p-value
Model 3	Night	0-50% (ref.)	-	-
		50-70%	1.01 [0.92-1.10]	0.85
		70-90%	1.15 [1.06-1.25][a]	0.0015
		90-100%	1.21 [1.08-1.35][a]	0.00064
	Day	0-50% (ref.)	-	-
		50-70%	0.90 [0.83-0.99][a]	0.031
		70-90%	0.84 [0.76-0.93][a]	0.00075
		90-100%	0.83 [0.72-0.94][a]	0.0042
Model 3 + short sleep duration	Night	0-50% (ref.)	-	-
		50-70%	0.99 [0.91-1.09]	0.90
		70-90%	1.11 [1.02-1.22][a]	0.019
		90-100%	1.11 [0.99-1.24]	0.075
	Day	0-50% (ref.)	-	-
		50-70%	0.91 [0.83-1.00]	0.051
		70-90%	0.84 [0.76-0.93][a]	0.00099
		90-100%	0.84 [0.73-0.96][a]	0.011
Model 3 + long sleep duration	Night	0-50% (ref.)	-	-
		50-70%	1.02 [0.93-1.12]	0.69
		70-90%	1.17 [1.07-1.28][a]	0.00053
		90-100%	1.21 [1.09-1.36][a]	0.00065
	Day	0-50% (ref.)	-	-
		50-70%	0.90 [0.82-0.99][a]	0.029
		70-90%	0.83 [0.74-0.91][a]	0.00023
		90-100%	0.81 [0.71-0.93][a]	0.0023
Model 3 + sleep efficiency	Night	0-50% (ref.)	-	-
		50-70%	1.02 [0.93-1.11]	0.73
		70-90%	1.16 [1.06-1.26][a]	0.0012
		90-100%	1.19 [1.06-1.33][a]	0.0022
	Day	0-50% (ref.)	-	-
		50-70%	0.90 [0.82-0.99][a]	0.032
		70-90%	0.83 [0.75-0.92][a]	0.0003
		90-100%	0.82 [0.72-0.94][a]	0.0034

[a]p<.05. Hazard ratios represent risks of all-cause mortality across light exposure percentile groups, compared to a reference light exposure group, for day and night light. Model 3 was adjusted for age, sex, ethnicity, photoperiod, employment status, education, income, deprivation, physical activity, smoking status, alcohol consumption, urbanicity, and social activity.

Table S7. Modeled circadian phase, amplitude, and mortality risk, adjusted for sleep duration and efficiency

		**All-cause mortality**	
		HR [95%CI]	p-value
Model 3	Mean Amplitude	0.96 [0.92-0.99][a]	0.0083
	Min. Amplitude	0.96 [0.93-0.99][a]	0.0052
	Max. Amplitude	0.94 [0.91-0.98][a]	0.0011
	Phase Variability	1.01 [0.98-1.03]	0.56
	Mean Phase		
	0-20%	1.16 [1.04-1.29][a]	0.0066
	20-40%	1.11 [0.99-1.24]	0.065
	40-60% (ref.)	-	-
	60-80%	1.07 [0.96-1.20]	0.22
	80-100%	1.13 [1.00-1.26][a]	0.042
Model 3 + short sleep duration	Mean Amplitude	0.98 [0.94-1.01]	0.19
	Min. Amplitude	0.98 [0.95-1.01]	0.27
	Max. Amplitude	0.94 [0.91-0.98][a]	0.0034
	Phase Variability	1.00 [0.98-1.03]	0.71
	Mean Phase		
	0-20%	1.17 [1.05-1.30][a]	0.0054
	20-40%	1.12 [1.00-1.26][a]	0.042
	40-60% (ref.)	-	-
	60-80%	1.09 [0.97-1.22]	0.16
	80-100%	1.12 [1.00-1.26][a]	0.048
Model 3 + long sleep duration	Mean Amplitude	0.95 [0.92-0.99][a]	0.0064
	Min. Amplitude	0.96 [0.93-0.99][a]	0.0053
	Max. Amplitude	0.93 [0.90-0.97][a]	0.00038
	Phase Variability	1.01 [0.98-1.04]	0.52
	Mean Phase		
	0-20%	1.19 [1.06-1.32][a]	0.0023
	20-40%	1.13 [1.01-1.26][a]	0.039
	40-60% (ref.)	-	-
	60-80%	1.09 [0.97-1.22]	0.16
	80-100%	1.14 [1.02-1.28][a]	0.023
Model 3 + sleep efficiency	Mean Amplitude	0.96 [0.92-0.99][a]	0.0088
	Min. Amplitude	0.96 [0.93-0.99][a]	0.011
	Max. Amplitude	0.93 [0.90-0.97][a]	0.00035
	Phase Variability	1.01 [0.98-1.03]	0.58
	Mean Phase		
	0-20%	1.18 [1.06-1.32][a]	0.0025
	20-40%	1.13 [1.01-1.26][a]	0.04
	40-60% (ref.)	-	-
	60-80%	1.09 [0.97-1.22]	0.16
	80-100%	1.14 [1.02-1.28][a]	0.027

[a]p<.05. Hazard ratios represent difference in mortality hazard per standard deviation increase in each circadian metric for mean, min., and max. amplitude, and phase variability. Hazard ratios for mean phase represent hazard of each percentile group relative to the 40-60% reference group, centered at the population mean phase (03:50). Phase ranges relative to sample mean for each percentile group were: -12 to -1.16 h (0-20%), -1.16 to -0.40 h (20-40%), 0.40 to 1.16 h (60-80%), and 1.16 to 12 h (80-100%). Model 3 was adjusted for age, sex, ethnicity, photoperiod, employment status, education, income, deprivation, physical activity, smoking status, alcohol consumption, urbanicity, and social activity.

Table S8. UK Biobank protocol documents

Document	Link
Invitation to participate	https://biobank.ctsu.ox.ac.uk/crystal/refer.cgi?id=100253
Death registry linkage	https://biobank.ctsu.ox.ac.uk/crystal/refer.cgi?id=115559
Light / accelerometer: Participant instructions	https://biobank.ndph.ox.ac.uk/showcase/refer.cgi?id=141141
Light / accelerometer: Collection and processing	https://biobank.ndph.ox.ac.uk/showcase/refer.cgi?id=131600
Assessment centre: Consent	https://biobank.ndph.ox.ac.uk/showcase/refer.cgi?id=100230
Assessment centre: Reception	https://biobank.ndph.ox.ac.uk/showcase/ukb/docs/Reception.pdf
Assessment centre: Blood pressure	https://biobank.ndph.ox.ac.uk/showcase/refer.cgi?id=100225
Assessment centre: Blood biochemistry	https://biobank.ndph.ox.ac.uk/showcase/refer.cgi?id=5636<br>https://biobank.ndph.ox.ac.uk/showcase/refer.cgi?id=1227

Table S9. UK Biobank variables

Variable	Collection method	UKB ID	Description	Link
Age	Registry	21003	Participant age (years) at assessment centre visit. Data obtained from NHS Primary Care Trust registries. Confirmed with participants at assessment centre, and amended if required.	https://biobank.ndph.ox.ac.uk/showcase/field.cgi?id=21003
Sex	Registry	31	Obtained from NHS Primary Care Trust registries. Confirmed with participants at assessment centre, and amended if required.	https://biobank.ndph.ox.ac.uk/showcase/field.cgi?id=31
Ethnic background	Assessment centre visit: Questionnaire	21000	Ethnic group (white, mixed, Asian/Asian British, black/black British, Chinese, other, PNTA) and ethnic background (sub-categories within each ethnic group).	https://biobank.ndph.ox.ac.uk/showcase/field.cgi?id=21000
Current employment status	Assessment centre visit: Questionnaire	6142	Paid employment, unemployed, retired, home/family caretaker, unable to work, volunteer, student, other	https://biobank.ndph.ox.ac.uk/showcase/field.cgi?id=6142
Average total household income before tax	Assessment centre visit: Questionnaire	738	Income brackets: <£18,000, £18,000-£29,900, £30,000-£51,900, £52,000-£100k,000, >£100,000, DNK, PNTA	https://biobank.ndph.ox.ac.uk/showcase/field.cgi?id=738
Townsend deprivation index	Registry	189	Scores represent average home ownership, car ownership, household overcrowding, and employment rate across each participant's postcode. Calculated using national census data at time of recruitment.	https://biobank.ndph.ox.ac.uk/showcase/field.cgi?id=189
Qualifications	Assessment centre visit: Questionnaire	6138	Qualifications obtained: University, A Levels, O Levels, NVQ/HND/HNC, CSE, other, none, PNTA	https://biobank.ndph.ox.ac.uk/showcase/field.cgi?id=6138
Leisure / social activities	Assessment centre visit: Questionnaire	6160	Participation in one or more weekly social activities, including: sports club /gym, pub or social club, adult education, other group activity, none, PNTA	https://biobank.ndph.ox.ac.uk/showcase/field.cgi?id=6160
Frequency of friend/family visits	Assessment centre visit: Questionnaire	1031	Frequency of visits from family/friends: Almost daily, 2-4 times a week, once a week, once a month, once every few months, never/almost never, no friends/family outside household, DNK, PNTA	https://biobank.ndph.ox.ac.uk/showcase/field.cgi?id=1031
Smoking status	Assessment centre visit: Questionnaire	20116	Smoking status / history: never, previous, current, PNTA	https://biobank.ndph.ox.ac.uk/showcase/field.cgi?id=20116
Alcohol intake frequency	Assessment centre visit: Questionnaire	1558	Typical alcohol consumption: Daily, 3-4 times per week, 1-2 times per week, 1-3 times per month, special occasions only, never, PNTA	https://biobank.ndph.ox.ac.uk/showcase/field.cgi?id=1558
Urbanicity	Registry	20118	'Urban' (population ≥ 10,000) and 'non-urban' (population < 10,000), defined according to population density of participant's postcode, derived from the UK Office for National Statistics.	https://biobank.ndph.ox.ac.uk/showcase/field.cgi?id=20118

Shift work	Assessment centre visit: Questionnaire	826	'Does your work involve shift work?' Answers: never, sometimes, usually, always, DNK, PNTA	https://biobank.ndph.ox.ac.uk/showcase/field.cgi?id=826
Night shift work	Assessment centre visit: Questionnaire	3426	'Does your work involve night shifts?' Answers: never, sometimes, usually, always, DNK, PNTA	https://biobank.ndph.ox.ac.uk/showcase/field.cgi?id=3426
Diabetes status	Assessment centre visit: Questionnaire	2443	'Has a doctor ever told you that you have diabetes?' Answers: Yes, no, DNK, PNTA	https://biobank.ndph.ox.ac.uk/showcase/field.cgi?id=2443
Vascular condition diagnosed by a doctor	Assessment centre visit: Questionnaire	6150	'Has a doctor ever told you that you have had any of the following conditions?' Answers: Heart attack, angina, stroke, high blood pressure, none, PNTA	https://biobank.ndph.ox.ac.uk/showcase/field.cgi?id=6150
Body mass index	Assessment centre visit: Physical	21001	Weight (kg) / height (m)^2	https://biobank.ndph.ox.ac.uk/showcase/field.cgi?id=21001
High density lipoprotein	Assessment centre visit: Physical	30760	Blood biochemistry assay	https://biobank.ndph.ox.ac.uk/showcase/field.cgi?id=30760
Low density lipoprotein	Assessment centre visit: Physical	30780	Blood biochemistry assay	https://biobank.ndph.ox.ac.uk/showcase/field.cgi?id=30780
Triglycerides	Assessment centre visit: Physical	30870	Blood biochemistry assay	https://biobank.ndph.ox.ac.uk/showcase/field.cgi?id=30870
Systolic blood pressure	Assessment centre visit: Physical	4080	Two readings, recorded at the start and end of assessment centre visit	https://biobank.ndph.ox.ac.uk/showcase/field.cgi?id=4080
Diastolic blood pressure	Assessment centre visit: Physical	4079	Two readings, recorded at the start and end of assessment centre visit	https://biobank.ndph.ox.ac.uk/showcase/field.cgi?id=4079
Physical activity	Accelerometer	90012	Axivity AX3 device average acceleration across one week data collection	https://biobank.ndph.ox.ac.uk/showcase/field.cgi?id=90012

DNK = 'do not know'; PNTA = 'prefer not to answer'

Table S10. Covariates included in statistical models, as derived from UK Biobank variables

Covariate	UKB ID(s)	Description
Age	21003	Strata, split by quartiles: Q1 [43.3-56.0], Q2 [56.0-63.4], Q3 [63.4-68.7], Q4 [68.7-82.0] (years at light/actigraphy recording)
Sex	31	Binary, male = 1, female = 0
Ethnicity	21000	Binary, white ethnic group = 1, other ethnic group = 0
Photoperiod	-	Continuous, calculated as the interval between sunrise and sunset at coordinates of 53.4808° N, 2.2426° W (Manchester), on date of light/accelerometer recording. Calculated using ‘getSunlightTimes()’ in the ‘suncalc’ package in R.
Physical activity	90012	Strata, split by quartiles: Q1 [0-21.9], Q2 [21.9-26.8], Q3 [26.8-32.4], Q4 [32.4-69.4] (milli-gravity)
Employment status	6142	Binary, employed = 1, other categories = 0
Income	738	Categorical: referent category = <£18k, £18k-£29.9k, £30k-£51.9k, £52k-£100k, >£100k, and ‘unknown’
Deprivation	189	Continuous, included as recorded
Education	6138	Categorical: ‘University’, ‘other non-university’ (any education category except ‘University’), referent category = ‘none’
Social activities	6160	Binary, >0 weekly activities = 1, 0 weekly activities = 0
Social visits	1031	Ordered categories: ‘no friends / family’ = 1 ‘never or almost never’ = 2, ‘every few months’ = 3, ‘monthly’ = 4, ‘weekly’ = 5, ‘2-4 times a week’ = 6, and ‘almost daily’ = 7
Smoking status	20116	Categorical, referent category = 'never'
Alcohol	1558	Continuous, representing days per week consuming alcohol. ‘Daily’ = 7, ‘3-4 times per week’ = 3.5, ‘1-2 times per week’ = 1.5, ‘1-3 times per month’ = 2*12/365.25*7, ‘special occasions only’ = 1*12/365.25*7, ‘never’ = 0
Urbanicity	20118	Binary, urban = 1, rural = 0
Shift work	826, 3426, 6142	Binary: shift-worker = 1, non-shift worker = 0. ‘Sometimes’, ‘usually’ or 'always' for ‘shift work’ and/or ‘night shift work’ = 1, ‘never/rarely’ = 0. Participants defined as ‘unemployed’ were defined as non-shift workers, using ‘Employment status’.
Diabetes status	2443	Binary strata, 'Yes' = 1, 'No' = 0
Vascular diagnosis	6150	Binary strata, ‘Heart attack’, and/or ‘Stroke’, and/or ‘Angina’, 'None' = 0
BMI	21001	Binary strata, BMI > 30 = 1, BMI ≤ 30 = 0
Cholesterol ratio	30760, 30780, 30870	Binary strata, ‘high’ = cholesterol ratio > 3.75 for males or > 3.00 for females. Calculated as cholesterol ratio = (HDL + LDL + 0.2*triglycerides)/HDL
Hypertension	4080, 4079	Binary strata, ‘high’ = systolic > 140 or diastolic > 90, where systolic and diastolic were included as the average of two readings (mmHg)

Table S11. STROBE Checklist

STROBE Statement—Checklist of items that should be included in reports of ***cohort studies***

	Item No	Recommendation	Manuscript Section
**Title and abstract**	1	(*a*) Indicate the study's design with a commonly used term in the title or the abstract	• Title
		(*b*) Provide in the abstract an informative and balanced summary of what was done and what was found	• Abstract
**Introduction**			
Background/rationale	2	Explain the scientific background and rationale for the investigation being reported	• Introduction: paragraphs 1-3
Objectives	3	State specific objectives, including any prespecified hypotheses	• Introduction: paragraph 4
**Methods**			
Study design	4	Present key elements of study design early in the paper	• Abstract<br>• Introduction: paragraph 4
Setting	5	Describe the setting, locations, and relevant dates, including periods of recruitment, exposure, follow-up, and data collection	• Abstract<br>• Introduction, Paragraph 4<br>• Results: paragraphs 1-2<br>• Materials and Methods: Light exposure: data collection; Cause-specific mortality; Covariates<br>• Section S1<br>• Table S8
Participants	6	(*a*) Give the eligibility criteria, and the sources and methods of selection of participants. Describe methods of follow-up	• Methods: Overview<br>• Materials and Methods: Light exposure: data collection; Cause-specific mortality<br>• Section S1<br>• Table S8
		(*b*) For matched studies, give matching criteria and number of exposed and unexposed	• N/A
Variables	7	Clearly define all outcomes, exposures, predictors, potential confounders, and effect modifiers. Give diagnostic criteria, if applicable	• Results: paragraph 1-3<br>• Materials and Methods (all sections except "Statistical Analysis")<br>• Tables S9-10

Data sources/ measurement	8*	For each variable of interest, give sources of data and details of methods of assessment (measurement). Describe comparability of assessment methods if there is more than one group	• Results: paragraph 1-3<br>• Materials and Methods (all sections except "Statistical Analysis")<br>• Tables S9-10
Bias	9	Describe any efforts to address potential sources of bias	• Materials and Methods: Statistical analysis<br>• Section S1
Study size	10	Explain how the study size was arrived at	• N/A
Quantitative variables	11	Explain how quantitative variables were handled in the analyses. If applicable, describe which groupings were chosen and why	• Results: Paragraphs 1-3<br>• Materials and Methods: Light exposure profiles; Circadian rhythm modeling<br>• Section S1<br>• Tables S9-10
Statistical methods	12	(*a*) Describe all statistical methods, including those used to control for confounding	• Materials and Methods: Statistical analysis<br>• Section S1
		(*b*) Describe any methods used to examine subgroups and interactions	• Tables S2-3
		(*c*) Explain how missing data were addressed	• Materials and Methods: Light exposure profiles<br>• Section S1
		(*d*) If applicable, explain how loss to follow-up was addressed	• N/A
		(*e*) Describe any sensitivity analyses	• Tables S2, S3, S6, S7
**Results**			
Participants	13*	(a) Report numbers of individuals at each stage of study—eg numbers potentially eligible, examined for eligibility, confirmed eligible, included in the study, completing follow-up, and analysed	• Results: Paragraphs 1-2<br>• Methods: Light exposure: data collection<br>• Section S1
		(b) Give reasons for non-participation at each stage	• Materials and Methods: Light exposure: data collection<br>• Section S1
		(c) Consider use of a flow diagram	• N/A
Descriptive data	14*	(a) Give characteristics of study participants (eg demographic, clinical, social) and information on exposures and potential confounders	• Results: paragraph 3<br>• Results: Table 1
		(b) Indicate number of participants with missing data for each variable of interest	• Materials and Methods: Light exposure: data collection; Light exposure profiles

			• Section S1
		(c) Summarise follow-up time (eg, average and total amount)	• Results, paragraph 2
Outcome data	15*	Report numbers of outcome events or summary measures over time	• Results, paragraph 2
Main results	16	(*a*) Give unadjusted estimates and, if applicable, confounder-adjusted estimates and their precision (eg, 95% confidence interval). Make clear which confounders were adjusted for and why they were included	• Results: Tables 2-3<br>• Tables S2, S3, S6, S7<br>• Methods: Covariates<br>• Section S1
		(*b*) Report category boundaries when continuous variables were categorized	• Table S10
		(*c*) If relevant, consider translating estimates of relative risk into absolute risk for a meaningful time period	• N/A
Other analyses	17	Report other analyses done—eg analyses of subgroups and interactions, and sensitivity analyses	• Tables S2, S3, S6, S7
**Discussion**			
Key results	18	Summarise key results with reference to study objectives	• Discussion: paragraphs 1,2,3,4
Limitations	19	Discuss limitations of the study, taking into account sources of potential bias or imprecision. Discuss both direction and magnitude of any potential bias	• Discussion: paragraph 8
Interpretation	20	Give a cautious overall interpretation of results considering objectives, limitations, multiplicity of analyses, results from similar studies, and other relevant evidence	• Discussion: paragraphs 2,3,4,9
Generalisability	21	Discuss the generalisability (external validity) of the study results	• Discussion: paragraph 8
**Other information**			
Funding	22	Give the source of funding and the role of the funders for the present study and, if applicable, for the original study on which the present article is based	• N/A
